# Phytochemistry and Biological Profile of *Gaultheria procumbens* L. and Wintergreen Essential Oil: From Traditional Application to Molecular Mechanisms and Therapeutic Targets

**DOI:** 10.3390/ijms25010565

**Published:** 2024-01-01

**Authors:** Piotr Michel, Monika Anna Olszewska

**Affiliations:** Department of Pharmacognosy, Faculty of Pharmacy, Medical University of Lodz, Muszyńskiego 1, 90-151 Lodz, Poland; piotr.michel@umed.lodz.pl

**Keywords:** *Gaultheria procumbens*, American wintergreen, eastern teaberry, wintergreen oil, phytochemistry, traditional use, biological activity

## Abstract

*Gaultheria procumbens* L. is a medicinal plant whose aerial parts (leaves, stems, and fruits) and methyl salicylate-rich essential oil (wintergreen oil) are used in phytotherapy to treat inflammation, muscular pain, and infection-related disorders. This overview summarises the current knowledge about ethnobotany, phytochemistry, pharmacology, molecular mechanisms, biocompatibility, and traditional use of *G. procumbens* and the wintergreen oil distilled from different plant organs. Over 70 hydrophilic compounds, including methyl salicylate glycosides, flavonoids, procyanidins, free catechins, caffeoylquinic acids, and simple phenolic acids, have been identified in *G. procumbens* plant parts. Moreover, aliphatic compounds, triterpene acids, and sterols have been revealed in lipophilic fractions. Furthermore, over 130 volatile compounds have been detected in wintergreen oil with dominating methyl salicylate (96.9–100%). The accumulated research indicates that mainly hydrophilic non-volatiles are responsible for the pharmacological effects of *G. procumbens*, primarily its potent anti-inflammatory, antioxidant, and photoprotective activity, with mechanisms verified in vitro and ex vivo in cellular and cell-free assays. The biological effectiveness of the dominant methyl salicylate glycoside—gaultherin—has also been confirmed in animals. Wintergreen oil is reported as a potent anti-inflammatory agent exhibiting moderate antioxidant and antimicrobial activity in vitro and significant insecticidal and larvicidal capacity. Together, *G. procumbens* accumulate a diverse fraction of polyphenols, triterpenes, and volatiles with validated in vitro and ex vivo biological activity but with the absence of in vivo studies, especially clinical trials concerning effective dose determination and toxicological verification and technological research, including drug formulation.

## 1. Introduction

**Figure 1:**
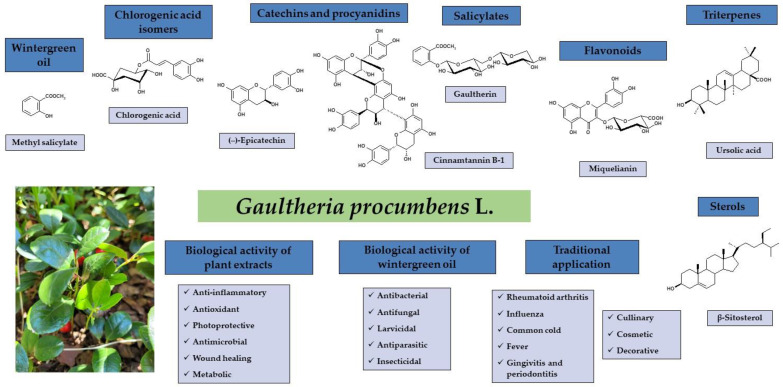
Summary of the chemical composition, biological activity, and traditional application of *G. procumbens* and wintergreen oil (picture taken by authors).

*Gaultheria procumbens* L. (American wintergreen, eastern teaberry), belonging to the Ericaceae family, is a small, low-growing shrub native to northeastern North America [1]. The plant has been used for hundreds of years in traditional medicine to treat disorders connected with inflammation or infection, especially rheumatoid arthritis, influenza, the common cold, and fever [2,3]. Historical reports on the chemical composition date back to the second half of the 19th century and relate mainly to methyl salicylate as the dominant component of *G. procumbens* essential oil (wintergreen oil) [4,5,6,7,8]. Later phytochemical research allowed the identification of over 70 bioactive constituents classified as simple phenolic acids, chlorogenic acid isomers, methyl salicylate glycosides, flavonoids, and proanthocyanidins among polyphenols, as well as triterpene acids and sterols [2]. The biological activity studies were mainly focused on the anti-inflammatory, antioxidant, and photoprotective activity of the plant extracts [2,3] as well as antimicrobial potential, such as antibacterial, antifungal, insecticidal, and larvicidal activities of the wintergreen oil [9,10]. *G. procumbens* is also of great industrial importance due to its culinary, cosmetic, and decorative qualities since it is a frequent food flavouring additive, a cosmetic ingredient of skin care products, and a popular ground cover plant [11,12,13], as shown in Figure 1.

The present review aims to summarise over 180 years of phytochemical and biological research on *G. procumbens* and methyl salicylate-rich wintergreen oil regarding their significance as anti-inflammatory, antioxidant, photoprotective, and antimicrobial agents. This work covers botanical description, phytochemical composition, pharmacological activity studies, molecular mechanisms, biocompatibility, and traditional application of different plant parts of eastern teaberry, primarily leaves and fruits—most commonly used in ethnomedicine, but also less popular stems and aerial parts. Furthermore, the chemical profile, pharmacological activity, and toxicology of wintergreen oil obtained from different organs of *G. procumbens* were reviewed. Eventually, the future perspectives and conditions of using extracts and essential oils for medicinal purposes and to prevent common diseases were discussed.

## 2. Botanical Description

### 2.1. Botanical Systematics

**Figure 2:**
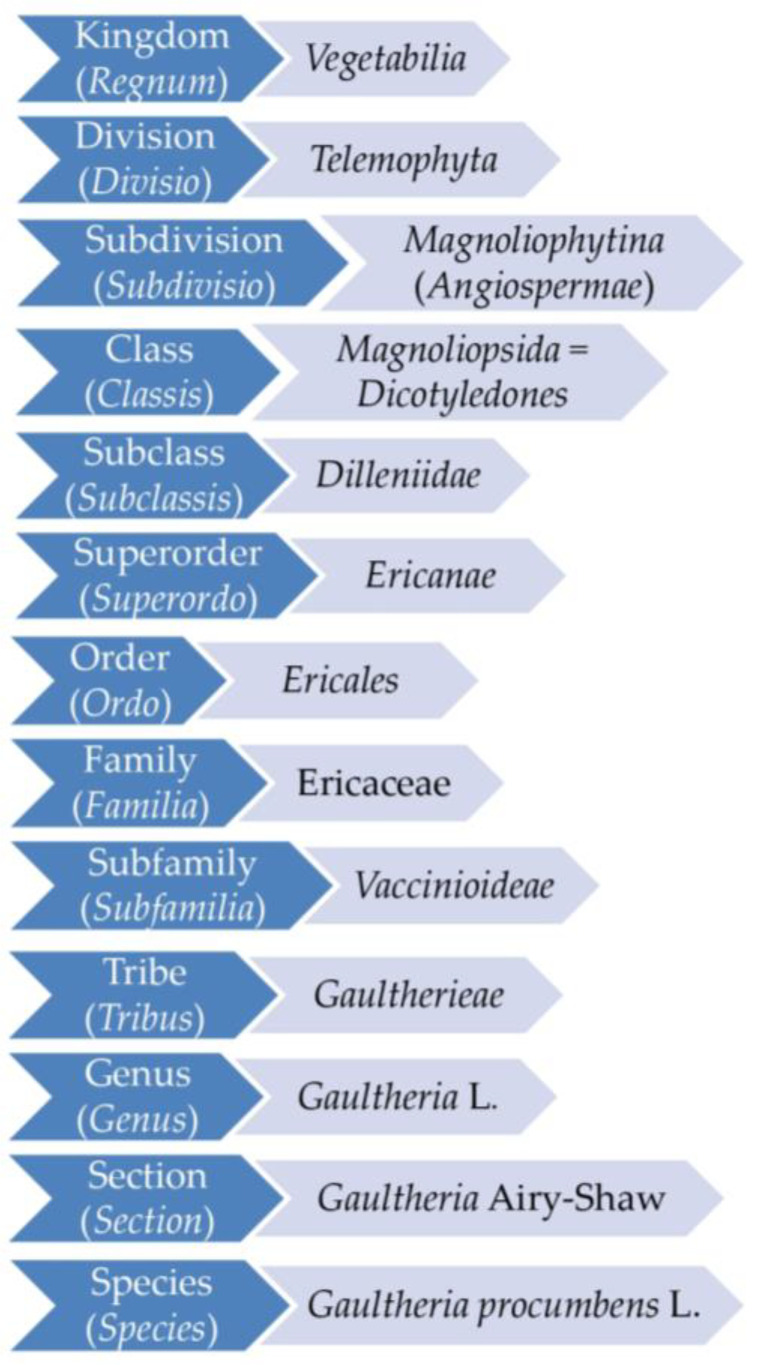
The present taxonomic rank of *G. procumbens*.

The genus *Gaultheria* L. (Ericaceae), widely distributed in the Americas, East Asia, and Oceania, has about 150 species. The complete taxonomic position of the genus and the species *G. procumbens*, following the latest phylogenetic research [14,15,16,17], is presented in Figure 2.

Historically, the first classification of the genus *Gaultheria*, dating from the mid-18th century, was introduced by Linnaeus [18], guided solely by the characteristics of the fruit. Later taxonomic approaches are often based on variations in numerous morphological elements within the genus, such as the shape of leaves or the structure of flowers and fruits [19,20,21,22]. Eventually, the detailed morphological and genetic studies at the end of the 20th century [23,24,25] allowed the proposal of a consistent, systematic classification currently accepted in science, according to which the genus *Gaultheria* covers 10 sections and 22 series [1].

### 2.2. Botanical Characteristics

**Figure 3:**
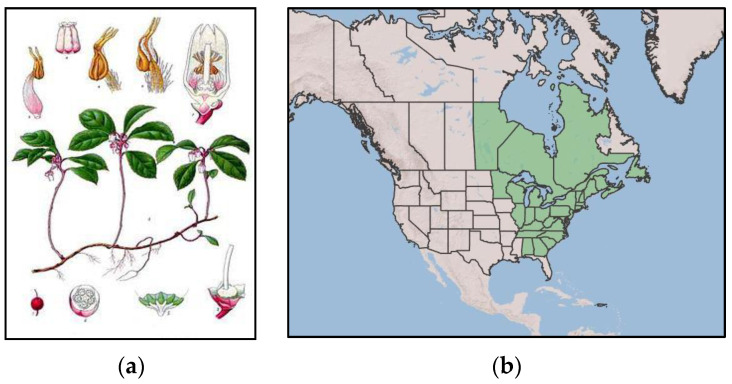
*Gaultheria procumbens* L.: (**a**) whole plant; (**b**) area of natural occurrence in North America marked in green [34].

*Gaultheria procumbens* L. (syn. *Brossaea procumbens* (L.) Kuntze) is known as American wintergreen, checkerberry, or eastern teaberry, according to the World Flora Online Plant List [26]. It is a small, prostrate, creeping shrub growing up to 20 cm in height (Figure 3a) [1,27,28,29,30,31,32]. American wintergreen occurs naturally in North America, from western Canada to the southeastern part of the United States (Figure 3b), and it prefers acidic soils. Under favourable conditions, it quickly colonises new areas, creating numerous stolons. It is commonly cultivated as an ornamental plant in Europe due to its decorative value and high frost resistance [12,27,29,32,33].

**Figure 4:**
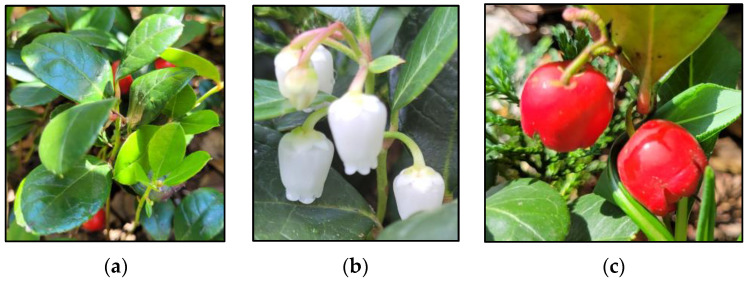
*Gaultheria procumbens* L.: (**a**) leaves; (**b**) flowers; (**c**) fruits (pictures taken by authors).

The leaves of *G. procumbens* are shiny, dark green, with pinnate innervation. The leaf blade is ellipsoidal and has an entire margin (Figure 4a). Young leaves, initially brown, turn green with time, while older leaves turn red in autumn [1,35,36]. Flowers of *G. procumbens* are pendulous, pentamerous in structure, solitary, or gathered in small clusters of 2–3 flowers growing in the axils of the leaves (Figure 4b) [1,29,30]. The fruit is a multi-seeded, spherical, slightly flattened capsule surrounded by fleshy calyx petals, intensely red in the colour of the outer pericarp, and white flesh with a characteristic smell of methyl salicylate (Figure 4c) [1,16,29,30,32].

The flowering time, depending on the area of occurrence, falls in May–September [29] or July–August [30,32], while the fruiting period is from early autumn to late summer of the following year [29,30,32].

## 3. Phytochemical Composition of *Gaultheria procumbens* and Wintergreen Oil

Among more than 150 species of the genus *Gaultheria*, the phytochemical profile and biological activity of only a few species, including *G. procumbens* and several representatives of Asian flora, have been relatively studied in detail so far. The analyses of the chemical composition of *G. procumbens* started in the second half of the 19th century and initially concerned only the essential oil as a source of methyl salicylate [6,7,8,37]. From the nineties of the 20th century, there was a systematic increase in interest in the profile of secondary metabolites of eastern teaberry, and the number of phytochemical studies concerning, in particular, leaves, stems, and fruits increased.

### 3.1. Phenols and Polyphenols

#### 3.1.1. Methyl Salicylate and Its Glycosidic Derivatives

**Figure 5:**
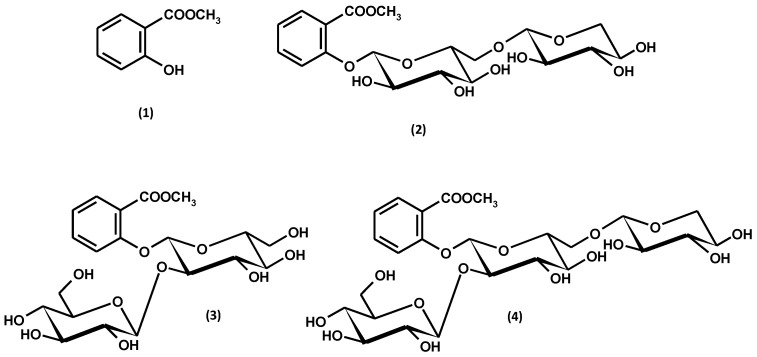
Methyl salicylate (**1**) and its glycosidic derivatives: gaultherin (**2**), physanguloside A (**3**), and 2-*O*-β-D-glucopyranosylgaultherin (**4**).

**Figure 6:**
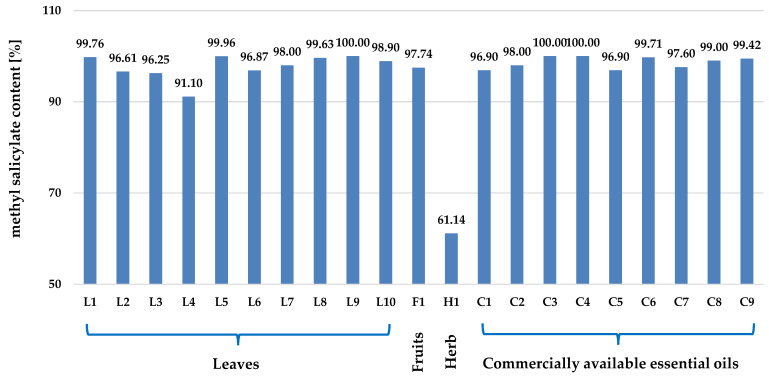
The content of methyl salicylate in essential oils distilled from leaves (L1–L10), fruits (F1), and herbs (aerial parts, H1) of *G. procumbens* and in commercially available *G. procumbens* essential oils (C1–C9). References: L1 [10], L2 [39], L3 [40], L4 [41], L5 [42], L6 [43], L7 [44], L8 [45,46], L9 [47], L10 [48], F1 [10], H1 [49], C1 [9], C2 [50], C3 [51], C4 [52], C5 [53], C6 [54], C7 [55], C8[56], C9 [57,58].

*G. procumbens*, classified as an essential oil-bearing plant, is characterised by a significant essential oil content, which is 1.30% dry weight (dw) for leaves and 2.68% dw for fruits, respectively [10]. The dominant component of the essential oil is methyl salicylate (Figure 5), constituting almost 99% of the entire complex, as shown in Figure 6 and Table S1. A similar high contribution of methyl salicylate is observed for essential oils obtained from other *Gaultheria* species. Therefore, all *Gaultheria* essential oils, regardless of the plant organs and species used for distillation, are called “wintergreen oils” [38].

Methyl salicylate glycosides in *G. procumbens* are represented by gaultherin (methyl salicylate 2-*O*-β-D-xylopyranosyl-(1→6)-β-D-glucopyranoside), 2-*O*-β-D-glucopyranosylgaultherin, and physanguloside A (Figure 5). Gaultherin and 2-*O*-β-D-glucopyranosylgaultherin were found in all organs of *G. procumbens*, including leaves, fruits, stems, and the whole aerial parts [59,60,61,62]. On the other hand, the presence of physanguloside A was revealed only in the fruits [61] and leaves [63]. Physanguloside A is a rare natural compound isolated so far only from *G. procumbens* and *Physalis angulata* [63]. The occurrence of free methyl salicylate, gaultherin, and other salicylic glycosides in *G. procumbens* is summarised in Table 1.

The first quantitative study of salicylates in various eastern teaberry organs concerned only the total salicylic acid content assayed by GC-MS following complete hydrolysis of the methanolic extracts. The results indicated the total salicylic acid level of 6.4 μg/g fresh weight (fw) in flowers, 3.8 μg/g fw in leaves, 2.2 μg/g fw in stems, 1.5 μg/g fw in fruits, 5.8 μg/g fw in whole wild plants, and 10.7 μg/g fw in whole cultivated plants [64]. In addition, the free salicylic acid, extracted directly with methanol from the over- and underground plant parts, reached 1.9 μg/g fw and 1.7 μg/g fw in wild and cultivated plants, respectively [64].

Further quantitative studies revealed methyl salicylate glycosides as the most significant fraction of polyphenols in eastern teaberry fruits and leaves and the most considerable polyphenolic constituents in stems, along with catechins and procyanidins [59,60,61]. It has been shown that the most efficient solvent for the extraction of salicylates from *G. procumbens* is acetone for stems [59] and fruits [61] and methanol–water (75:25, *v*/*v*) for leaves [60]. The high salicylate contents were determined by the HPLC-PDA method in the extracts prepared with these two solvents. Up to 199.9 mg/g of salicylates were detected in the stem dry extracts, among which gaultherin dominated (up to 185.9 mg/g) [59]. In the dry leaf and fruit extracts, the content of salicylates reached up to 187.5 mg/g and 121.7 mg/g, respectively, and this time gaultherin also prevailed, constituting up to 89–100% of the salicylate contents [60,61].

Reports indicate that gaultherin, the dominant methyl salicylate glycoside in *G. procumbens*, can be selectively isolated using fractionated extraction. It has been shown that with an increase in the polarity of the extractant, i.e., diethyl ether, ethyl acetate, and *n*-butanol, the content of gaultherin in the extract boosts. For instance, fractionation increased the concentration of gaultherin in the *n*-butanol fraction by 170% compared to the crude methanol–water extract of aerial parts (127.7 mg/g dw vs. 75.7 mg/g dw) [62].

#### 3.1.2. Phenolic Acids

The qualitative profile of *G. procumbens* phenolic acids does not differ significantly from that described for other representatives of the genus *Gaultheria* [2,65]. Available literature data indicated the presence of cinnamic acid derivatives common in the plant world, including caffeic and *p*-coumaric acids, as well as benzoic acid derivatives, such as *p*-hydroxybenzoic, protocatechuic and vanillic acids (Figure S1, Table 2). Unlike the extraction of salicylates, ethyl acetate was the best solvent for extracting phenolic acids from the plant material. The dry extracts from leaves, stems, and fruits prepared with this extractant contained up to 20.3 mg/g [60], 13.9 mg/g [59], and 6.0 mg/g [61] of phenolic acids analysed by HPLC-PDA, respectively. The predominant phenolic acids were isomeric monocaffeoylquinic acids, including chlorogenic (5-*O*-caffeoylquinic acid), neochlorogenic (3-*O*-caffeoylquinic acid), and cryptochlorogenic (4-*O*-caffeoylquinic acid) acids (Figure S2, Table 2). The total content of chlorogenic acid isomers, determined by HPLC-PDA, constituted up to 13–68% of the total phenolic acid levels [59,60,61]. The nomenclature of caffeoylquinic acid isomers is according to IUPAC [66].

#### 3.1.3. Flavonoids

**Figure 7:**
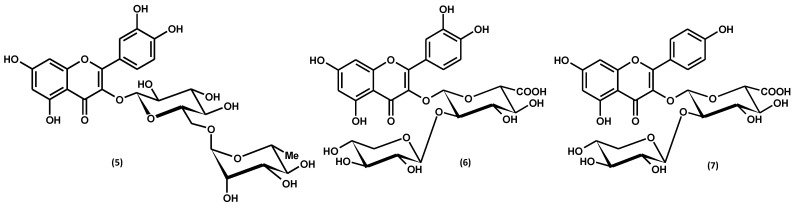
Flavonoid diglycosides: rutoside (rutin) (**5**), wintergreenoside A (**6**), and wintergreenoside B (**7**).

Flavonoid aglycones, i.e., quercetin and kaempferol, belonging to the class of flavonols, were identified in the hydrolysates from aerial parts of *G. procumbens* in the early 1990s [65]. Further analyses of the non-hydrolysed extracts revealed only traces of the two aglycones and the prevailing glycosides. A total of 14 flavonoid glycosides, including 11 monoglycosides and 3 diglycosides, have been identified in the plant, indicating a significant structural diversity of the *G. procumbens* flavonoid fraction, as shown in Figure 7 and Figure S3 and Table 3.

Most of the flavonoids of *G. procumbens* are common in nature, such as monoglycosides: hyperoside, isoquercitrin, miquelianin, astragalin, quercitrin, guaijaverin, or kaempferol-3-*O*-glucuronide, and a diglycoside–rutin [2,59,60,61,62,67,68]. On the other hand, two flavonol diglycosides containing glucuronic acid units in the sugar moiety, namely wintergreenosides A and B, have been found up to now only in this plant species [60,62]. As specific flavonoids of *G. procumbens*, they have been proposed as chemotaxonomic markers of the species. So far, only one flavonoid diglycoside of a similar structure (with a uronic acid unit in the glycone moiety) was found in the *Gaultheria* genus, i.e., quercetin 3-*O*-β-D-galacturonopyranosyl-(1→2)-β-D-glucopyranoside (dhasingreoside), isolated from stems and leaves of *G. fragrantissima* [69].

Regarding quantitative levels, flavonoids constitute the third fraction of *G. procumbens* polyphenols. It has been shown that methanol–water (75:25, *v*/*v*) is the best solvent for recovering this group of polyphenolic compounds from the plant material. The research revealed that the dry extracts from leaves, stems, and fruits prepared with this extractant contained up to 49.4 mg/g [60], 20.6 mg/g [59], and 1.2 mg/g [61] of flavonoids determined by the HPLC-PDA method, respectively. The dominant flavonoid of *G. procumbens* is miquelianin (quercetin 3-*O*-β-D-glucuronopyranoside), constituting about 65–85% of the flavonoid fraction, regardless of the plant organ [59,60,61].

#### 3.1.4. Catechins and Procyanidins

**Figure 8:**
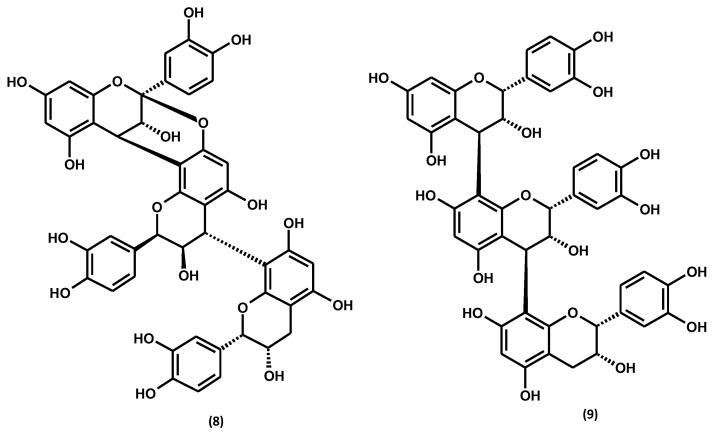
Procyanidin trimers: cinnamtannin B1 (**8**) and procyanidin C1 (**9**).

Like the rest of the *Gaultheria* species, *G. procumbens* biosynthesises flavan-3-ol derivatives with varying degrees of polymerisation [2]. Among them, 2 catechin monomers have been identified in the plant, including (+)-catechin and (−)-epicatechin, and 12 dimers and 8 procyanidin trimers, with dominating procyanidin B2, cinnamtannin B1, and procyanidin C1, as shown in Figure 8 and Figure S4 and Table 4. The flavan-3-ol monomers (+)-catechin and (−)-epicatechin and the dimeric procyanidin B2 are widespread among members of the genus *Gaultheria* [2,70]. However, the procyanidin A-type trimer, cinnamtannin B1, and procyanidin C1 are specific for *G. procumbens.*

Similarly to salicylates, it has been shown that the most efficient solvent for the extraction of catechins and procyanidins is acetone for stems [59] and fruits [61] and methanol–water (75:25, *v*/*v*) for leaves [60]. The total proanthocyanidin contents, determined by the spectrophotometric method for dry extracts prepared with these extractants, reached up to 174.4 mg/g for leaves [60], 241.6 mg for stems [59], and 62.4 mg/g for fruits [61]. The levels of low-molecular-weight procyanidins determined by the HPLC-PDA method constituted about 23–83% of the procyanidin levels determined by the spectrophotometric method [59,60,61], which revealed the co-occurrence of highly polymerised homologs in all plant organs of eastern teaberry. The HPLC-PDA quantitative profiling led to identifying (−)-epicatechin, procyanidin B2, and cinnamtannin B1 as the dominant flavan-3-ol derivatives of *G. procumbens*, which sum was, on average, 44–76% of the procyanidin contents [59,60,61].

#### 3.1.5. Other Phenolic and Polyphenolic Compounds

**Figure 9:**
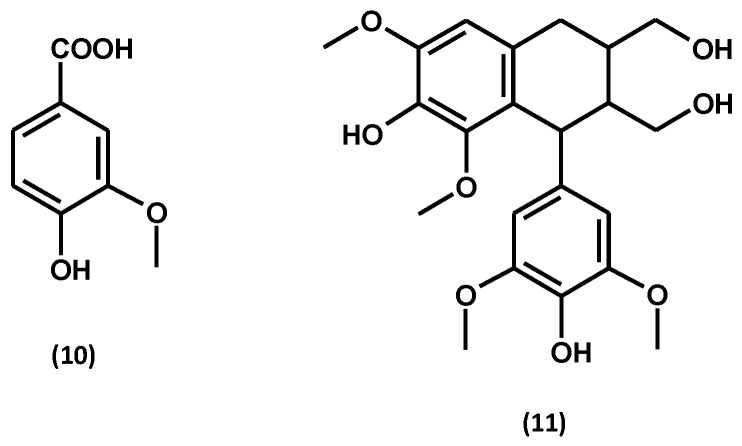
Other phenolic and polyphenolic compounds: vanillin (**10**) and lyoniresinol (**11**).

Apart from the compounds described above, leaves of *G. procumbens* contain vanillin [2,71], while lyoniresinol hexoside (an arylotetralin lignan) occurs in stems [59]. Their structures are shown in Figure 9.

#### 3.1.6. Total Phenolic Contents

In addition to quantitative levels of individual groups of polyphenols in *G. procumbens*, the literature sources also provide the total phenolic contents (TPC) examined by the Folin–Ciocalteu spectrophotometric method and expressed in gallic acid equivalents (mg GAE/g). The first reports concerned the TPC levels in fresh fruits extracted with methanol–water (8:2, *v*/*v*) and water acidified with citric acid, which were 1.3 mg GAE/g fw and 1.8–4.9 mg GAE/g fw, respectively [30,72]. The later studies of eastern teaberry fruits revealed the TPC values of 4.9 mg GAE/g fw and 7.7 mg GAE/g fw for the water and methanol–water (7:3, *v*/*v*) extracts, respectively [61].

As demonstrated for individual groups of compounds, methanol–water, and acetone were the best extractants of *G. procumbens* polyphenols [59,60,61,67,73]. Further quantitative analyses of dry extracts prepared with these solvents revealed TPC values reaching up to 302.4 mg GAE/g for leaves [60], 347.8 mg/g for stems [59], and 79.7 mg/g for fruits [61].

**Table 1:** Methyl salicylate and salicylic glycosides in *G. procumbens*.

Plant Part	Compound	Identification Method	Extract/Content	References
leaves	gaultherin (GT)	UV, TLC	−	[74]
	methyl salicylate (SM)	GC-MS	content in %: PE; SM: 2.31 CHE; SM: 6.88	[75]
	gaultherin (GT)	LC-MS	content in mg/g fw of the leaves: GT: 26.00 TSAL: 10.70	[76]
	gaultherin (GT)	UHPLC-PDA-ESI-MS^3^ HPLC-PDA	ME; GT mg/g dw of the leaves: 76.86 (April); 64.59 (May); 65.89 (June); 77.20 (July); 88.31 (August); 104.09 (September); 107.49 (October)	[67]
		isolation; UV, IR	−	[65,77]
	gaultherin (GT) physanguloside (PH)	isolation; ^1^H NMR; ^13^C NMR; 2D NMR; LC-MS/MS	water extract	[63]
	gaultherin (GT) gaultherin isomer 2-*O*-β-D-glucopyranosylgaultherin (TG)	UHPLC-PDA-ESI-MS^3^ HPLC-PDA	ME; GT: 98.41 mg/g dw of the extract	[62]
			content in mg/g dw of the extract: ME; TSAL: 98.89; GT: 98.41; TG: 0.49 EAE; TSAL: 288.13; GT: 288.13; TG: - BE; TSAL: 128.61; GT: 127.81; TG: 0.79	[60]
stems	gaultherin (GT)	UHPLC-PDA-ESI-MS^3^ HPLC-PDA	content in mg/g dw of the extract: ME; TSAL: 96.84; GT: 93.76 EAE; TSAL: 160.86; GT: 148.08 BE; TSAL: 152.71; GT: 138.61 AE; TSAL: 199.94; GT: 185.98 WE; TSAL: 19.88; GT: 10.52	[59]
			ME; GT: 93.76 mg/g dw of the extract	[62]
fruits	gaultherin (GT) physanguloside (PH) 2-*O*-β-D-glucopyranosylgaultherin (TG)	isolation; HR-ESI-MS, ^1^H NMR; ^13^C NMR; 2D NMR; identification of aglycone (GC-MS) and sugars (acid hydrolysis, HPLC-PDA)	content in mg/g dw of the extract: ME; TSAL: 83.66; GT: 48.89; PH: 12.45; TG: 22.31 EAE; TSAL: 109.28; GT: 98.25; PH: 8.58; TG: 2.45 BE; TSAL: 63.77; GT: 29.35; PH: 6.61; TG: 27.81 AE; TSAL: 121.67; GT: 93.63; PH: 16.09; TG: 11.95 WE; TSAL: 31.09; GT: 2.73; PH: 7.77; TG: 20.59	[61]
aerial parts	gaultherin (GT) GT isomer physanguloside (PH) 2-*O*-β-D-glucopyranosylgaultherin (TG)	UHPLC-PDA-ESI-MS^3^ HPLC-PDA	content in mg/g dw of the extract: ME; GT: 96.51 MED; GT: 75.68; DEF; GT: 21.37; EAF; GT: 30.25; BF; GT: 127.69; WF; GT: 27.81	[62]
−	gaultherin (GT)	−	−	[78,79]
−	methyl salicylate (SM) gaultherin (GT)	−	−	[80]

GT: methyl salicylate 2-*O*-β-D-xylopyranosyl-(1→6)-β-D-glucopyranoside (gaultherin); PH: methyl salicylate 2-*O*-β-D-glucopyranosyl-(1→2)-β-D-glucopyranoside (physanguloside A); TG: methyl salicylate 2-*O*-β-D-glucopyranosyl-(1→2)-[*O*-β-D-xylopyranosyl-(1→6)]-*O*-β-D-glucopyranoside; PE: petroleum ether dry extract; CHE: chloroform dry extract; MEC/ME: methanol–water dry extract (75:25, *v*/*v*) obtained by direct extraction of the raw material with a solvent; MED: defatted methanol–water extract (75:25, *v*/*v*) obtained by preliminary extraction of the raw material with chloroform in a Soxhlet apparatus, followed by extraction with a methanol–water solution (75:25, *v*/*v*); DEF: diethyl ether fraction (fractionated extraction); EAF: ethyl acetate fraction (fractionated extraction); BF: *n*-butanol fraction (fractionated extraction); WR/WF: water residue/fraction (fractionated extraction); ME: methanol–water dry extract (75:25, *v*/*v*); EAE: ethyl acetate dry extract (direct extraction); BE: *n*-butanol dry extract (direct extraction); AE: acetone dry extract (direct extraction); WE: water dry extract (direct extraction); TSAL: total salicylate content determined by HPLC-PDA-fingerprint (mg/g dw of the extract); dw: dry weight; fw: fresh weight.

**Table 2:** Phenolic acids in *G. procumbens*.

Plant Part	Compound	Identification Method	Extract/Content	References
derivatives of cinnamic and p-hydroxybenzoic acids				
leaves	*p*-hydroxybenzoic acid (*p*-HBA) protocatechuic acid (PCA) vanillic acid *p*-coumaric acid caffeic acid	UHPLC-PDA-ESI-MS^3^ HPLC-PDA	content in mg/g dw of the extract: MEC; TPHA: 8.86; SPHA: 1.81 MED; TPHA: 11.81; SPHA: 3.91 DEF; TPHA: 44.89; SPHA: 43.14 EAF; TPHA: 12.70; SPHA: 6.10 BF; TPHA: 14.01; SPHA: 3.05 WR; TPHA: 4.22; SPHA: 0.72	[68]
			ME; SPHA mg/g dw of the leaves: 1.13 (April); 1.12 (May); 1.02 (June); 0.93 (July); 0.99 (August); 0.98 (September); 0.87 (October)	[67]
	protocatechuic acid hexoside, protocatechuic acid (PCA), *p*-hydroxybenzoic acid (*p*-HBA)		content in mg/g dw of the extract: ME; TPHA: 11.31; SPHA: 4.51 EAE; TPHA: 20.33; SPHA: 14.45 BE; TPHA: 8.10; SPHA: 2.63	[60]
	*p*-hydroxybenzoic acid (*p*-HBA), protocatechuic acid (PCA), *o*-pyrocatechuic acid, syringic acid	−	−	[71]
	*p*-hydroxybenzoic acid (*p*-HBA), salicylic acid, *o*-pyrocatechuic acid, protocatechuic acid (PCA), vanillic acid, gentisic acid, *p*-coumaric acid, *o*-coumaric acid, caffeic acid	spectrophotometry and radioautography	ethanol–water extract (95:5, *v*/*v*) subjected to acid or alkaline hydrolysis	[81]
	*p*-coumaric acid	HPLC-DAD-APCI/MSD	ethanol–water extract (80:20, *v*/*v*)	[70]
	benzoic acid	−	−	[82]
	salicylic acid	TLC (gel plate, detection: Folin–Ciocalteu reagent)	ethyl acetate fraction obtained by extraction of plant material previously hydrolysed with 1 M HCl	[65]
stems	protocatechuic acid (PCA), derivatives of protocatechuic and caffeic acids, protocatechuic acid hexoside	UHPLC-PDA-ESI-MS^3^ HPLC-PDA	content in mg/g dw of the extract: ME; TPHA: 12.42; SPHA: 11.06 EAE; TPHA: 13.88; SPHA: 12.72 BE; TPHA: 12.03; SPHA: 10.49 AE; TPHA: 14.89; SPHA: 13.67 WE; TPHA: 11.91; SPHA: 10.57	[59]
fruits	*p*-hydroxybenzoic acid (*p*-HBA), protocatechuic acid (PCA), derivatives of vanillic, sinapinic, protocatechuic, and caffeic acids		content in mg/g dw of the extract: ME; TPHA: 2.25; PCA: 0.16; *p*-HBA: 0.08 EAE; TPHA: 6.03; PCA: 0.35; *p*-HBA: 0.22 BE; TPHA: 2.80; PCA: 0.43; *p*-HBA: 0.07 AE; TPHA: 3.23; PCA: 0.30; *p*-HBA: 0.21 WE; TPHA: 3.60; PCA: 0.27; *p*-HBA: 0.09	[61]
aerial parts	protocatechuic acid (PCA), protocatechuic acid hexosides		−	[62]
−	*o*-pyrocatechuic acid, gentisic acid, *p*-hydroxybenzoic acid (*p*-HBA), protocatechuic acid (PCA), vanillic acid, *p*-coumaric acid, caffeic acid, ferulic acid, salicylic acid, syringic acid	−	−	[77,80,83,84]
quinic acid derivatives, including monocaffeoylquinic acids				
leaves	3-*O*-*p*-coumaroylquinic acid 3-*O*-feruloylquinic acid 4-*O*-*p*-coumaroylquinic acid chlorogenic acid (CHA) neochlorogenic acid (NCHA) cryptochlorogenic acid (CCHA) caffeoylquinic acid derivative	UHPLC-PDA-ESI-MS^3^ HPLC-PDA	content in mg/g dw of the extract: MEC; TCHA: 7.05 MED; TCHA: 7.90 DEF; TCHA: 1.75 EAF; TCHA: 6.60 BF; TCHA: 11.05 WR; TCHA: 3.50	[68]
	3-*O*-*p*-coumaroylquinic acid chlorogenic acid (CHA) neochlorogenic acid (NCHA) cryptochlorogenic acid (CCHA) caffeoylquinic acid derivative		ME; TCHA mg/g dw of the leaves: 4.43 (April); 3.40 (May); 3.33 (June); 2.40 (July); 3.68 (August); 2.76 (September); 2.87 (October)	[67]
	chlorogenic acid (CHA) neochlorogenic acid (NCHA) cryptochlorogenic acid (CCHA)		ME; content in mg/g dw of the extract: NCHA: 3.68; CHA: 1.25; CCHA: 0.65	[62]
	3-*O*-*p*-coumaroylquinic acid derivative 3-*O*-*p*-coumaroylquinic acid hexoside chlorogenic acid (CHA) neochlorogenic acid (NCHA) cryptochlorogenic acid (CCHA) caffeoylquinic acid derivative		content in mg/g dw of the extract: ME; TCHA: 6.80; NCHA: 4.24; CHA: 1.68; CCHA: 0.88 EAE; TCHA: 5.88; NCHA: 2.89; CHA: 2.99; CCHA: - BE; TCHA: 5.47; NCHA: 2.91; CHA: 1.31; CCHA: 1.25	[60]
	chlorogenic acid isomer	HPLC-DAD-APCI/MSD	ethanol–water dry extract (80:20, *v*/*v*)	[70]
stems	3-*O*-*p*-coumaroylquinic acid derivative chlorogenic acid (CHA) neochlorogenic acid (NCHA)	UHPLC-PDA-ESI-MS^3^ HPLC-PDA	content in mg/g dw of the extract: ME; TCHA: 1.35 EAE; TCHA: 1.16 BE; TCHA: 1.53 AE; TCHA: 1.22 WE; TCHA: 1.34	[59]
	chlorogenic acid (CHA) neochlorogenic acid (NCHA) cryptochlorogenic acid (CCHA)		ME: content in mg/g dw of the extract: NCHA: 0.96; CHA: 0.96; CCHA: -	[62]
fruits	chlorogenic acid (CHA) neochlorogenic acid (NCHA)		content in mg/g dw of the extract: ME; CHA: 0.37 EAE; CHA: 0.59 BE; CHA: 0.35 AE; CHA: 0.76 WE; CHA: 0.40	[61]
aerial parts	3-*O*-*p*-coumaroylquinic acid derivative chlorogenic acid (CHA) neochlorogenic acid (NCHA) cryptochlorogenic acid (CCHA) caffeoylquinic acid derivative		content in mg/g dw of the extract: ME; NCHA: 3.68; CHA: 1.25; CCHA: 0.65 MED; NCHA: 2.48; CHA: 1.67; CCHA: 1.95 DEF; NCHA: 1.67; CHA: 2.46; CCHA: 0.81 EAF; NCHA: 5.68; CHA: 2.72; CCHA: 3.78 BF; NCHA: 6.47; CHA: 2.04; CCHA: 3.56 WF; NCHA: 2.34; CHA: 0.41; CCHA: 1.24	[62]

MEC/ME: methanol–water dry extract (75:25, *v*/*v*) obtained by direct extraction of the raw material with a solvent; MED: defatted methanol–water extract (75:25, *v*/*v*) obtained by preliminary extraction of the raw material with chloroform in a Soxhlet apparatus, followed by extraction with a methanol–water solution (75:25, *v*/*v*); DEF: diethyl ether fraction (fractionated extraction); EAF: ethyl acetate fraction (fractionated extraction); BF: *n*-butanol fraction (fractionated extraction); WR/WF: water residue/fraction (fractionated extraction); ME: methanol–water dry extract (75:25, *v*/*v*); EAE: ethyl acetate dry extract (direct extraction); BE: *n*-butanol dry extract (direct extraction); AE: acetone dry extract (direct extraction); WE: water dry extract (direct extraction); TPHA: total content of phenolic acids determined by HPLC-PDA-fingerprint (mg/g dw of the extract); SPHA: total content of simple phenolic acids derivatives of hydroxycinnamic and hydroxybenzoic acids determined by the HPLC-PDA-fingerprint (mg/g dw of the extract); TCHA: total monocaffeoylquinic acid content determined by HPLC-PDA-fingerprint (mg/g dw of the extract); dw: dry weight.

**Table 3:** Flavonoid aglycones and glycosides in *G. procumbens*.

Plant Part	Compound	Identification Method	Extract/Content	References
aglycones				
leaves	quercetin (QU) kaempferol (KA)	UHPLC-PDA-ESI-MS^3^ HPLC-PDA	extracts after acid hydrolysis; content in mg/g dw of the extract: MEC; FLC: 16.80; QU: 15.40; KA: 1.43 MED; FLC: 23.30; QU: 21.71; KA: 1.65 DEF; FLC: 15.70; QU: 14.42; KA: 1.32 EAF; FLC: 39.50; QU: 36.21; KA: 3.31 BF; FLC: 39.20; QU: 36.32; KA: 2.91 WR; FLC: 2.00; QU: 1.91; KA: 0.10	[68]
			content in mg/g dw of the extract: MEC; TFL: 34.40; QU: - MED; TFL: 47.20; QU: 0.17 DEF; TFL: 29.01; QU: 5.87 EAF; TFL: 82.60; QU: 1.94 BF; TFL: 79.60; QU: - WR; TFL: 4.40; QU: -	
			ME after acid hydrolysis, content in mg/g dw of the leaves: FLC: 9.95; QU: 9.41; KA: 0.55 (April); FLC: 8.20; QU: 7.80; KA: 0.40 (May); FLC: 8.13; QU: 7.72; KA: 0.43 (June); FLC: 8.58; QU: 8.10; KA: 0.48 (July); FLC: 9.10; QU: 8.60; KA: 0.50 (August); FLC: 11.93; QU: 11.21; KA: 0.73 (September); FLC: 12.54; QU: 11.70; KA: 0.84 (October)	[67]
	−		ME; TFL in mg/g dw of the leaves: 23.55 (April); 22.82 (May); 18.97 (June); 19.51 (July); 20.15 (August); 26.96 (September); 27.87 (October)	
	quercetin (QU)		ME; QU: 0.22 mg/g dw of the extract	[62]
			content in mg/g dw of the extract: ME; TFL: 49.35; QU: 0.22 EAE; TFL: 18.59; QU: 0.43 BE; TFL: 24.28; QU: 7.07	[60]
	quercetin (QU) kaempferol (KA)	TLC (cellulose plate, detection: UV, UV + ammonia)	ethyl acetate fraction obtained by extraction of plant material previously hydrolysed with 1 M HCl	[65]
	8-demethylsideroxylin 8-demethyllatifolin	−	a wax epicuticular layer of the leaves	[85]
stems	quercetin (QU) kaempferol (KA)	UHPLC-PDA-ESI-MS^3^ HPLC-PDA	content in mg/g dw of the extract: ME; TFL: 20.63; QU: 0.10; KA: - EAE; TFL: 5.26; QU: 0.32; KA: 0.22 BE; TFL: 11.38; QU: 1.69; KA: 0.20 AE; TFL: 11.08; QU: 0.24; KA: 0.08 WE; TFL: 14.93; QU: 0.07; KA: -	[59]
	quercetin (QU)		ME; QU: 0.1 mg/g dw of the extract	[62]
fruits	quercetin (QU) kaempferol (KA)		content in mg/g dw of the extract: ME; TFL: 1.17; QU: 0.14 EAE; TFL: 0.065; QU: 0.031 BE; TFL: 0.66; QU: 0.52 AE; TFL: 0.74; QU: 0.16 WE; TFL: 0.33; QU: 0.021	[61]
aerial parts	quercetin (QU)		content in mg/g dw of the extract: ME; QU: 0.19 MED; QU: 0.48; DEF; QU: 8.68; EAF; QU: 5.36; BF; QU: 7.74; WF: QU: 0.07	[62]
glycosides				
leaves	QU pentosyl glucuronide, hyperoside (HY), isoquercitrin (IQ), miquelianin (MQ), guaijaverin (GV), QU derivative, KA 3-*O*-glucuronide (KG), QU 3-*O*-glucuronide methyl ester, KA 3-*O*-glucuronide methyl ester, astragalin (AG), QU *n*-butyl 3-*O*-pentosyl glucuronide, QU *n*-butyl 3-*O*-glucuronide, KA *n*-butyl ester 3-*O*-pentosyl glucuronide, KA *n*-butyl ester 3-*O*-glucuronide	UHPLC-PDA-ESI-MS^3^ HPLC-PDA	content in mg/g dw of the extract: MEC; MQ: 26.84; HY: 2.43 MED; MQ: 37.68; HY: 2.90 DEF; MQ: 4.92; HY: 5.78 EAF; MQ: 44.51; HY: 23.18 BF; MQ: 67.89; HY: 1.38 WR; MQ: 1.68; HY: -	[68]
	QU pentosyl glucuronide, hyperoside (HY), rutin (RT), isoquercitrin (IQ), miquelianin (MQ), KA pentosyl glucuronide, guaijaverin (GV), QU hexosyl-rhamnoside, quercitin (QCT), KA 3-*O*-glucuronide (KG), QU pentosyl-rhamnoside		ME	[67]
	hyperoside (HY), miquelianin (MQ), wintergreenoside A (DGQ)		content in mg/g dw of the extract: ME; HY: 5.01; MQ: 32.14; DGQ: 7.26	[62]
	wintergreenoside A (DGQ), hyperoside (HY), isoquercitrin (IQ), miquelianin (MQ), KA 3-*O*-glucuronide (KG), wintergreenoside B (DGK), guaijaverin (GV)		content in mg/g dw of the extract: ME; DGQ: 7.26; HY: 5.01 IQ: 1.16; MQ: 32.14; GV: 1.46 EAE: DGQ: -; HY: 9.46; IQ: 2.97; MQ: 1.55; GV: 4.17 BE; DGQ: 1.24; HY: 5.33; IQ: 1.10; MQ: 6.72; GV: 1.73	[60]
	QU 3-*O*-galactoside, QU 3-*O*-glucoside, QU 3-*O*-arabinoside	HPLC-DAD-APCI/MSD	ethanol–water extract (80:20, *v*/*v*)	[70]
stems	hyperoside (HY) isoquercitrin (IQ) miquelianin (MQ) guaijaverin (GV)	UHPLC-PDA-ESI-MS^3^ HPLC-PDA	content in mg/g dw of the extract: ME; HY: 1.04; IQ: 1.08; MQ: 17.54; GV: 0.88 EAE: HY: 0.83; IQ: 1.37; MQ: 1.31; GV: 1.22 BE; HY: 1.44; IQ: 1.12; MQ: 5.64; GV: 0.96 AE; HY: 1.64; IQ: 1.06; MQ: 6.34; GV: 1.71 WE; HY: 0.62; IQ: 0.96; MQ: 13.28; GV: -	[59]
	hyperoside (HY), miquelianin (MQ), wintergreenoside A (DGQ)		content in mg/g dw of the extract: ME; HY: 1.04; MQ: 17.54; DGQ: 2.16	[62]
fruits	hyperoside (HY), isoquercitrin (IQ), miquelianin (MQ), QU hexosyl-rhamnoside, quercitin (QCT), KA 3-*O*-glucuronide (KG)		content in mg/g dw of the extract: ME; MQ: 0.95 EAE: MQ: - BE; MQ: - AE; MQ: 0.52 WE; MQ: 0.29	[61]
aerial parts	hyperoside (HY), isoquercitrin (IQ), miquelianin (MQ), guaijaverin (GV), KA 3-*O*-glucuronide (KG), wintergreenoside A (DGQ), wintergreenoside B (DGK)		content in mg/g dw of the extract: ME; HY: 3.24; MQ: 27.75; DGQ: 5.13 MED; HY: 5.34; MQ: 30.38; DGQ: 3.90 DEF; HY: 4.30; MQ: 9.72; DGQ: 1.02 EAF; HY: 30.04; MQ: 36.90; DGQ: 0.40 BF; HY: 9.88; MQ: 72.74; DGQ: 5.05 WF; HY: -; MQ: 22.84; DGQ: 10.65	[62]

HY: quercetin 3-*O*-β-D-galactopyranoside; IQ: quercetin 3-*O*-β-D-glucopyranoside; MQ: quercetin 3-*O*-β-D-glucuronopyranoside; GV: quercetin 3-*O*-α-L-arabinopyranoside; KG: kaempferol 3-*O*-β-D-glucuronopyranoside; AG: kaempferol 3-*O*-β-D-glucopyranoside; RT: quercetin 3-*O*-β-D-rhamnopyranosyl-(1→6)-β-D-glucopyranoside; QCT: quercetin 3-*O*-α-L-rhamnopyranoside; DGQ: quercetin 3-*O*-β-D-xylopyranosyl-(1→2)-β-D-glucuronopyranoside; DGK: kaempferol 3-*O*-β-D-xylopyranosyl-(1→2)-β-D-glucuronopyranoside; MEC/ME: methanol–water dry extract (75:25, *v*/*v*) obtained by direct extraction of the raw material with a solvent; MED: defatted methanol–water extract (75:25, *v*/*v*) obtained by preliminary extraction of the raw material with chloroform in a Soxhlet apparatus, followed by extraction with a methanol–water solution (75:25, *v*/*v*); DEF: diethyl ether fraction (fractionated extraction); EAF: ethyl acetate fraction (fractionated extraction); BF: *n*-butanol fraction (fractionated extraction); WR/WF: water residue/fraction (fractionated extraction); ME: methanol–water dry extract (75:25, *v*/*v*); EAE: ethyl acetate dry extract (direct extraction); BE: *n*-butanol dry extract (direct extraction); AE: acetone dry extract (direct extraction); WE: water dry extract (direct extraction); FLC: total flavonoids determined by HPLC-PDA as sum of aglycones after acid hydrolysis (mg/g dw of the extract); TFL: total flavonoid content determined by HPLC-PDA-fingerprint (mg/g dw of the extract); dw: dry weight.

**Table 4:** Catechins and procyanidins in *G. procumbens*.

Plant Part	Compound	Identification Method	Extract/Content	References
catechins				
leaves	(+)-catechin (CA) (−)-epicatechin (ECA)	UHPLC-PDA-ESI-MS^3^ HPLC-PDA	content in mg/g dw of the extract: MEC; TPA: 189.87; TLPA: 108.70; ECA: 1.90 MED; TPA: 290.90; TLPA: 93.80; ECA: 8.88 DEF; TPA: 115.67; TLPA: 116.11; ECA: 2.31 EAE; TPA: 483.40; TLPA: 133.50; ECA: 75.40 BF; TPA: 441.87; TLPA: 133.50; ECA: - WR; TPA: 49.60; TLPA: 5.32; ECA: -	[68]
	(−)-epicatechin (ECA)		content in mg/g dw of the leaves: ME; TPA: 61.21; TLPA: 28.71; ECA: 7.67 (April); TPA: 57.90; TLPA: 24.23; ECA: 6.11 (May); TPA: 55.60; TLPA: 23.52; ECA: 6.14 (June); TPA: 53.03; TLPA: 22.83; ECA: 5.82 (July); TPA: 58.70; TLPA: 29.70; ECA: 6.98 (August); TPA: 66.74; TLPA: 30.27: ECA: 7.79 (September); TPA: 66.80; TLPA: 29.63; ECA: 7.38 (October)	[67]
			ME; ECA: 9.07 mg/g dw of the extract	[62]
			content in mg/g dw of the extract: ME; TPA: 174.38; TLPA: 63.99; ECA: 9.07 EAE; TPA: 36.93; TLPA: 9.67; ECA: 7.91 BE; TPA: 87.32; TLPA: 43.64; ECA: 0.54	[60]
	(+)-catechin (CA) (−)-epicatechin (ECA)	HPLC-DAD-APCI/MSD	ethanol–water (80:20, *v*/*v*) extract	[70]
stems	(−)-epicatechin (ECA)	UHPLC-PDA-ESI-MS^3^ HPLC-PDA	ME; ECA: 24.35 mg/g dw of the extract	[62]
	(+)-catechin (CA) (−)-epicatechin (ECA)		content in mg/g dw of the extract: ME; TPA: 240.17; TLPA: 126.77; ECA: 24.35 EAE; TPA: 51.60; TLPA: 69.68; ECA: 19.33 BE; TPA: 122.30; TLPA: 82.94; ECA: 13.01 AE; TPA: 241.61; TLPA: 201.31; ECA: 36.15 WE; TPA: 179.09; TLPA: 76.86; ECA: 12.47	[59]
fruits			content in mg/g dw of the extract: ME; TPA: 62.39; TLPA: 7.41; ECA: 2.36 EAE; TPA: 0.58; TLPA: -; ECA: - BE; TPA: 2.92; TLPA: -; ECA: - AE; TPA: 41.26; TLPA: 9.59; ECA: 1.57 WE; TPA: 30.01; TLPA: 1.24; ECA: 0.22	[61]
aerial parts	(−)-epicatechin (ECA)		content in mg/g dw of the extract: ME: ECA: 12.47 MED; ECA: 12.31 DEF; ECA: 0.31 EAF; ECA: 79.73 BF; ECA: - WF; ECA: -	[62]
procyanidins				
leaves	A-type dimers, B-type dimers, A-type trimers, B-type trimers, cinnamtannin B1 (CB1)	UHPLC-PDA-ESI-MS*^3^* HPLC-PDA	content in mg/g dw of the extract: MEC; CB1: 23.37 MED; CB1: 35.80 DEF; CB1: 4.30 EAE; CB1: 136.99 BF; CB1: 72.75 WR; CB1: 5.21	[68]
	A-type dimer, B-type trimer, procyanidin B2 (PB2), cinnamtannin B1 (CB1)		content in mg/g dw of the leaves: ME; PA: 6.60 (April); 5.50 (May); 5.62 (June); 6.94 (July); 8.56 (August); 8.61 (September); 9.53 (October)	[67]
	procyanidin B2 (PB2), cinnamtannin B1 (CB1)		content in mg/g dw of the extract: ME; PB2: 13.15; CB1: 18.61	[62]
	A-type dimer, B-type trimer, procyanidin B2 (PB2), procyanidin C1, cinnamtannin B1 (CB1)		content in mg/g dw of the extract: ME; PB2: 13.15; CB1: 19.61 EAE; PB2: -; CB1: 0.77 BE; PB2: 6.29; CB1: 12.89	[60]
	procyanidin B2 (PB2), procyanidin A2	HPLC-DAD-APCI/MSD	ethanol–water extract (80:20, *v*/*v*)	[70]
stems	A-type dimers, B-type dimers, A-type trimers, B-type trimers, procyanidin B2 (PB2), cinnamtannin B1 (CB1)	UHPLC-PDA-ESI-MS^3^ HPLC-PDA	content in mg/g dw of the extract: ME; PB2: 17.22; CB1: 25.11 EAE; PB2: 10.35; CB1: 15.18 BE; PB2: 11.41; CB1: 16.09 AE; PB2: 22.25; CB1: 30.57 WE; PB2: 11.58; CB1: 15.00	[59]
	procyanidin B2 (PB2), cinnamtannin B1 (CB1)		content in mg/g dw of the extract: ME; PB2: 17.22; CB1: 25.11	[62]
fruits	A-type dimers, B-type dimers, A-type trimers, B-type trimers, procyanidin B2 (PB2), cinnamtannin B1 (CB1), procyanidin C1		content in mg/g dw of the extract: ME; PB2: 2.86; CB1: 0.89 EAE; PB2: -; CB1: - BE; PB2: -; CB1: - AE; PB2: 2.28; CB1: 3.48 WE; PB2: 0.34; CB1: 0.68	[61]
aerial parts	A-type dimers, B-type dimers, A-type trimers, B-type trimers, procyanidin B2 (PB2), cinnamtannin B1 (CB1), procyanidin C1		content in mg/g dw of the extract: ME; PB2: 14.25; CB1: 19.84 MED; PB2: 13.46; CB1: 14.42 DEF; PB2: 6.12; CB1: - EAF; PB2: 35.05; CB1: 154.57 BF; PB2: 8.36; CB1: 15.51 WF; PB2: 2.10; CB1: -	[62]

PB2: epicatechin-(4β→8)-epicatechin; CB1: epicatechin-(4β→8, 2β→*O*→7)-epicatechin-(4β→8)-epicatechin; MEC/ME: methanol–water dry extract (75:25, *v*/*v*) obtained by direct extraction of the raw material with a solvent; MED: defatted methanol–water extract (75:25, *v*/*v*) obtained by preliminary extraction of the raw material with chloroform in a Soxhlet apparatus, followed by extraction with a methanol–water solution (75:25, *v*/*v*); DEF: diethyl ether fraction (fractionated extraction); EAF: ethyl acetate fraction (fractionated extraction); BF: *n*-butanol fraction (fractionated extraction); WR/WF: water residue/fraction (fractionated extraction); ME: methanol–water dry extract (75:25, *v*/*v*); EAE: ethyl acetate dry extract (direct extraction); BE: *n*-butanol dry extract (direct extraction); AE: acetone dry extract (direct extraction); WE: water dry extract (direct extraction); TPA: total proanthocyanidin content determined by *n*-BuOH/HCl spectrophotometric method; Porter’s method (mg/g dw of the extract); TLPA: total proanthocyanidin content determined by HPLC-PDA-fingerprint (mg/g dw of the extract); dw: dry weight.

The total levels of polyphenols in plants are routinely determined by the Folin–Ciocalteu method. However, in the case of *G. procumbens*, the accurate total and individual phenolic contents were better reflected by the results determined by high-performance techniques. The total phenolic content analysed by the HPLC-PDA method for dry extracts reached up to 336.7 mg/g for leaves [60], 427.2 mg/g for stems [59], and 135.2 mg/g for fruits [61], which were significantly higher than the corresponding TPC values by an average of 11–70%. This tendency was explained by the dominant contribution of methyl salicylate glycosides in the polyphenolic profiles and the low reactivity of salicylate aglycone in redox reactions [73].

### 3.2. Terpenoids

#### 3.2.1. Essential Oil Components

In addition to the main component, i.e., methyl salicylate, numerous simple, aliphatic hydrocarbons, alcohols, and carboxylic acids, as well as a group of monoterpenes, with dominating limonene, linalool, and pinenes, were identified in the essential oils obtained from different organs of *G. procumbens*. The structures of primary compounds and the qualitative and quantitative profiles of the essential oils are presented in Figure S5 and Table S1, respectively.

#### 3.2.2. Triterpenes and Sterols

Triterpenes in *G. procumbens* are represented mainly by derivatives of two basic structures, i.e., ursane and oleanane, among which several pentacyclic triterpene alcohols and acids have been found with dominant ursolic and oleanolic acids. Moreover, the literature data indicate the presence of phytosterols common in the plant world with prevailing β-sitosterol. So far, no glycosidic forms such as triterpene saponins have been reported. The structures of selected representatives of the triterpenes and sterols in *G. procumbens* and their quantitative profiles are presented in Figure S6 and Table 5.

The content of ursolic and oleanolic acids in *G. procumbens* was assayed by UHPLC-PDA and reached 4.55–7.07 mg/g dw in leaves [75] and 7.34 mg/g dw in the chloroform–methanol leaf dry extract (1:1, *v*/*v*) [86]. The main component of the fraction was ursolic acid, the content of which constituted 79–83% of the sum of triterpene acids.

**Table 5:** Triterpenes and sterols in *G. procumbens*.

Plant Part	Compound	Identification Method	Extract/Content	References
leaves	ursolic acid (UA) oleanolic acid (OA) α-amyrin β-amyrin β-sitosterol (β-SIT) campesterol	GC-MS	content in % *: PE; UA: 4.27; OA: 1.70; β-SIT: 2.68 CHE; UA: 28.82; OA: 10.11; β-SIT: -	[75]
	ursolic acid (UA) oleanolic acid (OA)	UHPLC-PDA	content in mg/g dw of the leaves: CHE **; OA: 1.11; UA: 4.55 (April); OA: 0.84; UA: 3.71 (May); OA: 0.99; UA: 4.23 (June); OA: 1.26; UA: 5.41 (July); OA: 1.14; UA: 5.36 (August); OA: 1.15; UA: 5.67 (September); OA: 1.20; UA: 5.87 (October)	
			content in mg/g dw of the extract: CHE **; OA: 1.58; UA: 5.76	[86]

UA: ursolic acid; OA: oleanolic acid; β-SIT: β-sitosterol; PE: petroleum ether dry extract; CHE: chloroform dry extract; * relative concentrations of compounds in the extracts according to the peak area ratio (%) observed in the total ion chromatograms; CHE **: chloroform–methanol dry extract (1:1, *v*/*v*), obtained after pre-extraction of the plant material with *n*-hexane.

### 3.3. Other Lipophilic Non-Volatile Compounds

**Figure 10:**
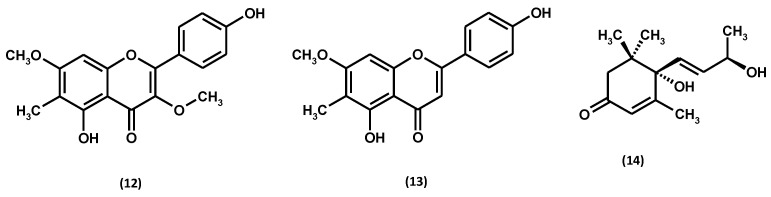
Lipophilic flavonoids and a norisoprenoide of *G. procumbens*: 8-demethyllatifolin (**12**), 8-demethylsideroxylin (**13**), and (1*S*,9*S*)-vomifoliol (**14**).

GC-MS analysis of petroleum ether and chloroform extracts from leaves of *G. procumbens* allowed the identification of several additional classes of lipophilic compounds, i.e., aliphatic hydrocarbons and alcohols as well as aliphatic and aromatic carboxylic acids [75]. Among the hydrocarbons, the following were identified: neophytadiene, tetracosan, pentacosan, heneicosane, heptacosan, hexacose-1-ene, squalene, octacosan, nonacosan, hexadecane, docosan, and tritriacontan. Aliphatic alcohols are represented by phytol, tetracosan-1-ol, hexacosan-1-ol, heptacosan-1-ol, octacosan-1-ol, and 4-hydroxyphenylethanol. The group of carboxylic acids included: dodecanoic acid (lauric acid), hexadecanoic acid (palmitic acid), octadecanoic acid (stearic acid), docosanoic acid (behenic acid), tetracosanoic acid (lignoceric acid), hexacosanoic acid (cerotic acid) and *m*-methoxybenzoic acid (*m*-anisic acid). Among the lipophilic compounds, the presence of flavonoid aglycones rarely found in the plant kingdom, i.e., 8-demethyllatifolin and 8-demethylsideroxylin, and a norisoprenoide (1*S*,9*S*)-vomifoliol was found (Figure 10). In addition, methyl benzoate, stigmastan-3,5-diene, α-tocopherol, and 13-docosamide (erucamide) have also been identified in the lipophilic leaf extracts of *G. procumbens* [75].

In the petroleum ether extract of *G. procumbens* leaves, a total of 32 compounds were identified by GC-MS, representing 86% of all analytes in the extract. Among them, waxy substances (48%) from the group of hydrocarbons, alcohols, and carboxylic acids dominated. Some waxy and terpenoid derivatives have also been identified in the chloroform extract of *G. procumbens* leaves. However, simple aliphatic hydrocarbons (6%), alcohols (3%), and carboxylic acids (6%) were this time in the minority compared to the other ingredients (67%), dominated by ursolic (29%) and oleanolic (10%) acids, as well as methyl benzoate (10%) and methyl salicylate (7%) [75].

### 3.4. Mineral Elements

The level of two macroelements (calcium and magnesium) and two microelements (iron and zinc) was measured by atomic absorption spectroscopy in dry extracts prepared with water acidified with citric acid from *G. procumbens* fruits harvested in three different fruit maturity stages. The content of Ca, Mg, Fe, and Zn reached up to 19.42 mg/100 g dw, 14.95 mg/100 g dw, 3.53 mg/100 g dw, and 1.69 mg/100 g dw, respectively. It has been shown that the Mg, Fe, and Zn levels in eastern teaberry extracts were independent of the fruit harvest date. On the other hand, the Ca level was strongly related to the harvesting time and was the highest in extract from fruits collected in the full maturity phase [30].

### 3.5. Seasonal Variability of the Chemical Composition

The qualitative and quantitative composition change during the growing season is a common plant feature [87]. Research has shown that the TPC levels and the total contents of procyanidins, salicylates, and phenolic acids with chlorogenic acid isomers (Table 1, Table 2, and Table 4) varied relatively narrowly for the leaves of *G. procumbens* harvested in the entire growing season, i.e., from April to October in monthly intervals, covering up to 15–25% of the highest concentrations. Only the variations of flavonoid contents (Table 3) and ursolic and oleanolic acid levels (Table 5) were more pronounced but remained relatively moderate, with differences within, at most, 35–37% of the maximal value. The high concentrations of polyphenolic constituents and triterpene acids of *G. procumbens* leaves were found to be biosynthesised in a relatively wide vegetation frame, indicating potential ease in choosing the time of collecting plant material. Eventually, the autumn months (September and October) were proposed as the optimal period for harvesting plant material in Polish climatic conditions [67,75].

## 4. Biological Activity of *Gaultheria procumbens* Extracts and Wintergreen Oil

**Figure 11:**
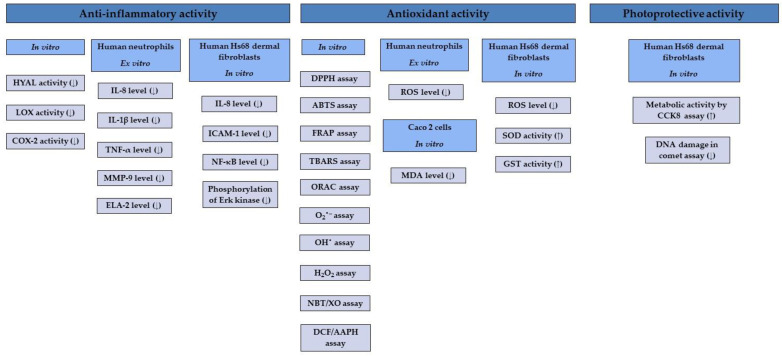
Molecular mechanisms of anti-inflammatory, antioxidant, and photoprotective activity of *G. procumbens*. (↑) upregulation; (↓) downregulation.

The therapeutic potential of *G. procumbens* was first discovered by the indigenous tribes of North America. The plant materials most often used in traditional phytotherapy are leaves and fruits, applied especially in the treatment of inflammation, including rheumatoid arthritis, diseases of the upper respiratory tract, colds, dermatoses, and pain of various aetiologies [2]. The first scientific reports on the medicinal use of *G. procumbens* came from the first half of the 19th century and concerned the anti-inflammatory, analgesic, and antipyretic effects of essential oil and leaf infusions [2,3,4,5]. Later in vitro and ex vivo studies focused mainly on anti-inflammatory, antioxidant, and photoprotective activity, their molecular mechanisms (Figure 11), and the potential antimicrobial and metabolic activity of extracts from various plant organs, especially leaves, fruits, stems, and whole aerial parts of eastern teaberry (leafy stems with flowers or fruits). The essential oils’ in vitro antioxidant and antimicrobial activity were also emphasised. Moreover, the biological effects of gaultherin—the primary salicylate constituent of *G. procumbens*—have been confirmed in vivo.

### 4.1. Biocompatibility and Toxicity

The long-term use of *G. procumbens* preparations indicates their general safety as a food ingredient and medical plant, and reports of some toxic effects only concern the ingestion of wintergreen oil. The cellular biocompatibility of plant extracts was confirmed in vitro using several cell lines. The accumulated research has shown that the leaf, stem, and fruit extracts (25–150 μg/mL) do not reduce the viability of human neutrophils, as documented by flow cytometry with propidium iodide staining [59,60,61]. The cellular safety of the ethanolic extract of *G. procumbens* leaves (5–100 μg/mL) towards human intestinal Caco-2 cells has also been proved by MTT assay [88]. No statistically significant reduction in metabolic activity of L929 mouse fibroblast by MTT reduction assay and viability of UVA unirradiated Hs68 human dermal fibroblasts by CCK-8 assay was also observed for eastern teaberry leaf, stem, and fruit extracts analysed at 0.5–100 μg/mL and 5–25 μg/mL, respectively [89]. Moreover, wintergreen oil (1.82–58.34 mg/mL) demonstrated no statistically significant reduction in peripheral blood mononuclear cell viability analysed by MTT assay. The essential oil also did not cause double-stranded DNA damage monitored by the PicoGreen^®^ dsDNA assay and did not induce DNA damage tested by the comet assay at 1.82–29.17 mg/mL and 1.82–3.64 mg/mL, respectively [42].

The therapeutic effect of plant materials rich in salicylates may be associated with the risk of side effects in vivo. In the case of *Gaultheria* plants, essential oil is the most severe cause of poisoning. Reported cases of poisoning were usually caused by oral ingestion, especially by children [50], or higher doses of essential oil absorption through the skin [90,91,92,93,94,95]. The first symptoms of “wintergreen oil” poisoning resemble contact dermatitis, in extreme cases leading to anaphylactic shock. The gastrointestinal tract becomes irritated, and the acid–base balance is disturbed, manifested by acidosis or metabolic alkalosis. Often, there are dizziness, visual and auditory disturbances, loss of consciousness, and convulsions. Severe poisoning leads to pulmonary oedema, acute renal failure, and coma. Accidental ingestion of as little as 4 mL in children and 6 mL in adults of wintergreen oil is already lethal [50]. Death is most often found as a result of respiratory loss and extensive damage to internal organs [50,96,97,98,99,100,101,102]. The overview of case reports concerning toxicity after accidental or intentional (suicide attempt) ingestion or topical application of wintergreen oil or its preparations is shown in Table 6.

A specific manifestation of the toxicity of wintergreen oil might be its anticancer activity. However, only a single literature report investigated such potential. It has been shown that the essential oil distilled from *G. procumbens* leaves (10–400 mg/L) tested on primary rat healthy neurons and N2a neuroblastoma cells by MTT reduction assay revealed weak cytotoxic activity only at high concentrations, thus presenting negligible anticarcinogenic activity against neuroblastoma cells [103]. 

Due to the high oral toxicity, wintergreen oil (applied in appropriate, relatively low concentrations) remains indicated only for external use for the relief of musculoskeletal pain and to treat influenza, fever, and the common cold [2].

### 4.2. Anti-Inflammatory Activity

The anti-inflammatory activity is the leading biological effect of *G. procumbens*, as suggested by traditional medicine [2,3], so most literature data have been devoted to this topic. Both hydrophilic extracts, reflecting in the best manner the composition of the overall plant matrix, and the most popular medicinal preparations, like tinctures and infusions [88], as well as compounds isolated from the plant material, were analysed to verify the anti-inflammatory effectiveness and molecular mechanisms of action of the plant preparations and determine the main active ingredients responsible for their effects.

It was shown that the leaf, stem, and fruit extracts of *G. procumbens* inhibited the three critical pro-inflammatory enzymes, i.e., hyaluronidase, lipoxygenase, and cyclooxygenase 2 in vitro (Table 7). Stem extracts showed the highest inhibitory activity towards hyaluronidase and lipoxygenase enzymes, and fruit extracts towards cyclooxygenase 2. Moreover, results revealed that methyl salicylate glycosides and procyanidins, as the dominant fractions of fruit and stem polyphenols, contribute primarily to the inhibitory activity of *G. procumbens* extracts towards pro-inflammatory enzymes [59,60,61,62,68].

**Table 6:** Case reports of the wintergreen oil poisoning.

Patient	Age *	Hospital	Drug Administration and Formulation **	Symptoms of Poisoning	Death/ Recovery ***	Year of the Event	References
male	−	−	accidental ingestion of wintergreen oil as a tea sweetener (30 mL)	−	(D)	1832	[104]
male	−	−	accidental ingestion of wintergreen oil (60 mL)	diarrhoea, sweating, internal burning, skin intensely red	(D)	1900	[105]
female	40Y	−	accidental ingestion of wintergreen oil (30 mL)	burning in the abdomen, extreme nausea, vomiting, diarrhoea, tinnitus	(R)	1918	[106]
male with severe cough and shortness of breath, former soldier, and alcoholic	25Y	−	accidental ingestion of wintergreen oil as a soda drink ingredient	vomiting, no history of any convulsion	(D)	1927	[107]
male infant	22M	−	accidental ingestion of wintergreen oil	vomiting, tonic seizures	(D)	1927	
43 patients: newborns, infants, children	1M-3.5Y	−	accidental ingestion of wintergreen oil (4–60 mL)	nausea, vomiting, fever, dehydration, electrolyte disturbance, tinnitus, haematologic disturbances, non-cardiogenic pulmonary oedema, seizures, coma	(R)/(D)	1937–1992	[50]
male child	2Y3M	−	accidental ingestion of wintergreen oil (30 mL)	burning of the mouth, vomiting, dyspnoea, paleness, dehydration, restlessness, twitching	(D)	1944	[108]
infant	−	Children’s National Hospital, District of Columbia, USA	accidental ingestion of wintergreen oil	−	(D)	1948	[109]
female infant with a cold	25M	Royal Hospital for Sick Children, Edinburgh, Scotland	accidental ingestion of wintergreen oil (less than 15 mL)	vomiting, loose stools, irritability, dyspnoea, paleness, muscle twitching	(D)	1953	[100]
male child	2Y	Salt Lake Country General Hospital, Salt Lake City, UT, USA	accidental ingestion of wintergreen oil (20 mL)	vomiting, hyperactivity; serum salicylate concentration (66 mg/100 mL on admission, 86 mg/100 mL after 2 h after admission, 51 mg/100 mL after blood transfusion, 6.7 mg/100 mL after 67 h after admission)	(R)	1956	[110]
male child	2Y	St. Francis Hospital, Blue Island, IL, USA	accidental ingestion of wintergreen oil (60 mL)	vomiting, tachypnoea, stupor; serum salicylate concentration (57.9 mg/100 mL after 12 h after admission, 54 mg/100 mL after 28 h after admission)	(R)	1957	[111]
84 patients: newborns, infants, children, males, females	1.2M-88Y	−	oral salicylate ingestion 86%; oral wintergreen oil (methyl salicylate) ingestion 11%; rectal salicylate 1%; topical salicylate 2%	altered consciousness (62%), seizures (11%), vomiting (22%), hyperthermia (43%), hypotension (15%), pulmonary oedema (5%), acute kidney injury (15%), median peak serum salicylate concentration 86 mg/dL (range 35–238 mg/dL)	(R)—88%; (D)—11%; permanent sequelae—1%	1958–2013	[112]
male child	3Y2M	Winnipeg Children’s Hospital, Canada	accidental ingestion of wintergreen oil (15 mL)	vomiting, heavy breathing, drowsiness, hyperventilation, dehydration; serum salicylate concentration (69 mg% on admission, 37 mg% after blood transfusion, 32 mg% 9 h after blood transfusion)	(R)	1960	[113]
male (Indian stoker) with a cold	−	cargo ship in the Red Sea	ingestion of wintergreen oil (60 mL) as a cold medicine	abdominal pain, board-like rigid of abdominal muscles, urticarial rash, itch	(R)	1968	[114]
three patients (sex unknown)	−	Mackay Memorial Hospital, Taipei, Taiwan	accidental ingestion of wintergreen oil	hyperventilation, high serum level of salicylate	(D)—1 patient; (R)—2 patients	1974–1976	[115]
female infant	21M	−	wintergreen oil in the form of candy flavouring	vomiting, lethargy, hyperpnea; serum salicylate concentration (81 mg/dL 6 h after ingestion)	(R)	1985	[116]
male	44Y	McLeod Regional Medical Centre, Florence, SC, USA	accidental ingestion of wintergreen oil (30 mL)	seizures, tachypnoea, diarrhoea, confusion, diaphoresis, cardiopulmonary arrest; serum salicylate concentration (78.3 mg/dL on admission)	(D)	1989	[117]
24 patients: males and females	15–86Y	Prince of Wales Hospital, Shatin, New Territories, Hong Kong, China	accidental ingestion (2 patients), suicidal attempt or as self-treatment for a stroke (1 patient), or self-poisoning (21 patients) of/with ‘White Flower Oil’ ^A^ (amount ingested: not known—11 patients, ≤10 mL—5 patients, 10–20 mL—2 patients) or ‘Red Flower Oil’ ^B^ (amount ingested: not known—2 patients, ≤10 mL—1 patient, 10–20 mL—1 patient, 30–100 mL—2 patients)	‘White Flower Oil’ ^A^ (severity of salicylate poisoning: no symptoms—7 patients, mild—11 patients, moderate–severe—0 patients); ‘Red Flower Oil’ ^B^ (severity of salicylate poisoning: no symptoms—0 patients, soft—3 patients, moderate–severe—3 patients); serum salicylate concentration: ‘White Flower Oil’ ^A^ (0.1–1.0 mM/L—12 patients, 1.1–2.1 mM/L—5 patients, ≥2.2 mM/L—1 patient); ‘Red Flower Oil’ ^B^ (0.1–1.0—2 patients, 1.1–2.1 mM/L—0 patient, ≥2.2 mM/L—4 patients)	‘White Flower Oil’ ^A^: (R)—18 patients, (D)—0 patients; ‘Red Flower Oil’ ^B^: (R)—5 patients, (D)—1 patient	1991–1998	[118]
female with hypertension and arthritis	70Y	The Poison Control Center Children Hospital of Philadelphia, Pennsylvania; Cooper Hospital/University Medical Center-UMDNJ/RWJ Camden, New York; Shore Memorial Hospital Somers Point, New York, USA	ingestion of Koong Yick Huang FA Oil^®^ (60 mL; containing 67% wintergreen oil) in an attempt to relieve chronic knee pain	abdominal pain, restlessness, diaphoresis, tachycardia, seizures, metabolic acidosis; serum salicylate concentration (108.6 mg/dL on admission)	(D)	1998	[102]
female infant	18M	Great Ormond Street Hospital, London, UK	accidental ingestion of wintergreen oil	nausea, vomiting, irritability, lethargy, tachypnoea; serum salicylate concentration (4.8 mmol/L on admission)	(R)	2001	[97]
male	40Y	Royal Prince Alfred Hospital Emergency Department, Sydney, Australia	wintergreen oil applied topically by an unregistered Chinese herbalist	vomiting, tinnitus, dizziness, erythema, sweating; serum salicylate concentration (48.5 mg/dL 36 h after ingestion)	(R)	2002	[96]
female	29Y	National Poisons Information Service, Birmingham; Edinburgh; Cardiff; Newcastle, UK	intentional ingestion of wintergreen oil (60 mL) with alcohol in a suicide attempt	cardiac arrest for 30 min; serum salicylate concentration (1196 mg/L 4 h after ingestion)	(D)	2003–2022	[119]
male	25Y		intentional ingestion of wintergreen oil (15 mL) over a 2 h period with alcohol (suicide attempt); intentional ingestion of wintergreen oil (15 mL) after 5 days	serum salicylate concentration (639 mg/L 2.5 h after ingestion); serum salicylate concentration (1150 mg/L 6.5 h after ingestion)	(R)		
patient (sex unknown)	−		intentional ingestion of wintergreen oil (35 mL) in a suicide attempt	serum salicylate concentration (629 mg/L at unknown time post-ingestion)	−		
patient (sex unknown)	−		intentional ingestion of wintergreen oil in a suicide attempt	serum salicylate concentration (661 mg/L 6 h after ingestion)	−		
female	79Y	Tondabayashi Hospital, Osaka, Japan	Teipap A Cool^®^ compress (Teikoku Seiyaku Co., Ltd., Kagawa, Japan) soaked with wintergreen oil	rectangular pruritic, erythematous macule on the hip	(R)	2004	[120]
male with diabetes mellitus, nephropathy, and coronary artery disease	80Y	San Francisco General Hospital, San Francisco, USA	accidental ingestion of wintergreen oil (mouthful volume ≈ 21 mL)	vomiting, tonic-clonic seizure, unresponsiveness, apnoea; serum salicylate concentration (74.8 mg/dL after dialysis, 82.6 mg/dL *post-mortem*)	(D)	2007	[121]
male infant	16M	Hospital Kulim, Kulim, Kedah, Malaysia	accidental ingestion of wintergreen oil as a massage oil	vomiting, tonic-clonic seizure, dehydration, hypotonia; serum salicylate concentration (112.15 mg/L on admission)	(R)	2012	[122]
male	32Y	National Hospital of Sri Lanka, Sri Lanka	accidental ingestion of wintergreen oil	sudden syncopal attack, tonic-clonic seizure, cardiac arrest	(D)	2015	[123]
male	42Y	National Hospital of Sri Lanka, Sri Lanka	accidental ingestion of wintergreen oil (10 mL)	vomiting, burning epigastric pain, anxiety, restlessness, tachypnoea, metabolic acidosis and respiratory alkalosis, tonic-clonic seizure	(R)	2015	
female with hypertension, asthma, and ischaemic cerebral stroke	77Y	Ewha Womans University Mokdong Hospital, Seoul, Korea	accidental ingestion of topical liniment (Mentholatum^®^ containing wintergreen oil)	hyperthermia, tachypnoea, metabolic acidosis; serum salicylate concentration (320 mg/L one day after admission)	(R)	2016	[124]
male child (12.6 kg)	2Y	Emergency Medicine Unit, The University of the West Indies, St. Augustine, Trinidad and Tobago	accidental ingestion of wintergreen oil	five episodes of vomiting, tremors, tachycardia, tachypnoea, tonic-clonic seizures, cardiac arrest	(R)	2016	[125]
female with hands, forearms, legs, and face dermatitis for the last two months	45Y	−	R.C.^TM^ essential oil blend (containing wintergreen oil) as aromatherapy for pain relief	erythema, itchy plaques, papules, and vesicles in the areas mentioned above	(R)	2020	[126]
female (43 kg weight) with depression, atrial fibrillation, and sleep apnoea	74Y	Utah Poison Control Centre, Salt Lake City, UT, USA	two cups of EcoLogic^TM^ Home Insect Control insecticide (containing wintergreen oil) in a suicide attempt	abdominal pain, vomiting, difficulty in hearing, tinnitus, tachypnoea; serum salicylate concentration (72.5 mg/dL 2 h after ingestion, 59 mg/dL at 6 h, 39.3 ng/dL at 18 h, 22.8 mg/dL at 30 h, 17 mg/dL at 38 h)	(R)	2020	[127]
male	67Y	Bordeaux University Hospital, Bordeaux, France	French green clay (Cattier, Paris, France; containing 10% wintergreen oil) topically once a day for three days as a leave-on product	a 3-week history of erythema and bullous lesions on the right knee	(R)	2022	[128]
male with diabetes mellitus for the past 10 years	58Y	Bhagwan Mahaveer Jain Hospital, Bengaluru, Karnataka, India	accidental ingestion of wintergreen oil (5–6 mL)	giddiness, uneasiness, restlessness, tachycardia, tachypnoea, metabolic acidosis, respiratory alkalosis, hypotension	(R)	2022	[129]

* age in months (M) or years (Y); ** wintergreen oil (containing min. 98% methyl salicylate); *** death (D) or recovery (R); ^A^ ‘White Flower Oil’ (containing 40% of methyl salicylate); ^B^ ‘Red Flower Oil’ (containing 30–67% of methyl salicylate, depending on the product brand).

Further studies demonstrated that *G. procumbens* leaf, stem and fruit extracts (25–150 μg/mL) significantly downregulated the release of pro-inflammatory cytokines, i.e., IL-8, IL-1β, and TNF-α, as well as tissue-remodelling enzymes, i.e., matrix metalloproteinase 9 and human elastase type 2 in a model of human neutrophils stimulated with bacterial lipopolysaccharide (LPS) or *N*-formyl-L-methionyl-L-leucyl-L-phenylalanine (*f*MLP) with cytochalasin B ex vivo (Table 7). The impact of the tested extracts on the pro-inflammatory function of human immune cells was the most significant for the secretion of IL-1β, ELA-2, and TNF-α [59,60,61]. Another study indicated that eastern teaberry leaf, stem, and fruit extracts (0.5–100 μg/mL) did not increase the THP1-Blue^TM^ NF-κB monocyte activity and did not induce NF-κB transcription factor in human Hs68 dermal fibroblasts, which proved the lack of their pro-inflammatory potential [89]. Simultaneously obtained results showed that plant extracts (5–50 μg/mL) substantially diminished the release of NF-κB, the expression of ICAM-1, and the secretion of IL-8 by LPS-stimulated Hs68 fibroblasts (Table 7) and suppressed (25–50 μg/mL) the LPS-induced Erk kinase activation in dermal cells [89]. All the mentioned mechanisms of anti-inflammatory activity confirm the validity of using *G. procumbens* in treating inflammation-related disorders connected with overexpression of the appropriate pro-inflammatory mediators. The most important is rheumatoid arthritis, which indeed is suggested by traditional medicine as the primary therapeutic target for eastern teaberry [2], just as skin inflammatory disorders, for instance, psoriasis [132,133,134].

The strong anti-inflammatory activity has also been proved for the dominant *G. procumbens* methyl salicylate glycoside, i.e., gaultherin, in both in vitro and ex vivo cellular assays and in vivo animal models. Zhang and others [135] documented that gaultherin (0.3–3.0 μg/mL) significantly inhibited the release of TNF-α, IL-6, and IL-1β from the LPS-stimulated RAW 264.7 murine macrophages. Olszewska and co-workers [62] confirmed the strong potential of gaultherin (25–75 μM) to diminish the secretion of IL-1β, TNF-α, and human elastase type 2 from the LPS-stimulated human neutrophils ex vivo. Eventually, Zhang and associates [136] showed that gaultherin (400 mg/kg), isolated from stems and leaves of *G. yunnanensis*, revealed substantial antinociceptive (acetic acid-induced abdominal contraction test) and anti-inflammatory (croton oil-induced ear oedema) activity in mice. Simultaneously, the results showed that gaultherin (100 mM; 330 mg/kg) did not cause damage in the gastric mucosa (determined by the average lesion score) and did not affect the ulcerogenic response to water immersion restraint stress in rats [136]. Zhang and others [137] also showed that salicylate-rich fraction (200–800 mg/kg; containing around 50% of gaultherin), isolated from *G. yunnanensis* stems and leaves, presented significant analgesic activity on acetic acid-induced writhing, formalin-induced pain, and hot plate tests in mice. At the same time, salicylate-rich fraction exhibited important anti-inflammatory activity on croton oil-induced ear oedema in mice and carrageenan-induced paw oedema in rats [137]. Alam and co-workers [138] demonstrated significant analgesic (acetic acid-induced writhing and tail immersion tests), anti-inflammatory (carrageenan- and croton oil-induced paw oedema models), and antipyretic (Brewer’s yeast-induced pyrexia assay) effect in mice of salicylate-rich fraction (100–300 mg/kg), obtained from stems and leaves of *G. trichophylla*. Regarding similar composition of salicylate fractions with dominating gaultherin in numerous *Gaultheria* species, all the accumulated data generally confirmed the essential contribution of gaultherin and salicylates to the anti-inflammatory effects of *Gaultheria* plants in vivo, including *G. procumbens*.

### 4.3. Antioxidant Activity

Oxidative stress, reflected by the overproduction of reactive oxygen species in cells, is strongly correlated with chronic inflammation [139]; therefore, antioxidant effects have also become a frequently explored research field for the plant extracts, wintergreen oil, and dominant active ingredients of *G. procumbens*. The results obtained in five complementary in vitro non-cellular tests, i.e., DPPH, ABTS, and FRAP, the three most commonly used methods based on the single electron transfer (SET), and the linolenic acid oxidation inhibition and peroxide radical scavenging tests, two procedures related to hydrogen atom transfer (HAT), revealed significant antioxidant activity for eastern teaberry leaf and stem extracts, especially when compared to the potential of standard antioxidants, both synthetic, such as Trolox^®^, or natural, such as quercetin or ascorbic acid (Table 7). It was also shown that leaf and stem extracts demonstrated substantial ability to scavenge in vivo-relevant radicals directly, e.g., O_2_^•−^, OH^•^ and H_2_O_2_, typically released in the inflammatory process [30,59,60,67,68,72,88,130]. Contrary to extracts from leaves and stems of eastern teaberry, fruit extracts showed relatively moderate direct antioxidant activity, which resulted straight from the domination of salicylic glycosides in the quantitative profile of *G. procumbens* fruits and their low reactivity in SET and HAT reactions [61].

Further research indicated that *G. procumbens* leaf, stem, and fruit extracts (25–150 μg/mL) significantly downregulated ROS production by *f*MLP-stimulated human neutrophils ex vivo, and the most potent activity was observed for leaf and stem extracts [59,60], what was explained by the high content of procyanidins and flavonoids with well-documented in vitro and in vivo antioxidant activity. Interestingly, the results also demonstrated a substantial decrease in the ROS level by the fruit extract [61]. What is also important is that compared to the positive control (quercetin), the cellular antioxidant potential of the fruit extract was considerably higher than its direct ROS scavenging ability noted in cell-free assays. This phenomenon was explained by the influence of methyl salicylate glycosides, abundant in the *G. procumbens* fruits, on the pro-inflammatory intracellular signalling pathways [140,141]. Voicu and others [88] also demonstrated the cellular antioxidant effects of the tincture (25–100 μg/mL) prepared from the leaves of *G. procumbens* with ethanol–water (90:10, *v*/*v*); the tincture reduced oxidative stress and lowered the level of lipid peroxidation monitored by the amount of the malondialdehyde in the Caco-2 cell line stimulated with H_2_O_2_.

Oxidative stress caused by high-dose UV radiation leads to cellular damage, e.g., extracellular matrix degradation, collagen loss, and elastin fibre denaturation, contributing to accelerated and premature skin ageing [142]. It was shown by the DCFH_2_-DA assay that *G. procumbens* leaf, stem, and fruit extracts (5–25 μg/mL) diminished the ROS generation in UVA-irradiated human Hs68 dermal fibroblasts [89]. The three extracts (5–25 μg/mL) also restored the activity of endogenous antioxidant enzymes superoxide dismutase and glutathione *S*-transferase in the UVA-irradiated Hs68 cells [89]. These results indicated that the antioxidant activity of eastern teaberry translates into its photoprotective effects.

The antioxidant activity studies in simple cell-free and cellular assays also concerned the wintergreen oil. The results obtained in simple chemical tests were quite discrepant, but in general, they suggest weak or, at most, moderate antioxidant effects of the oil. One exception is the work of Nikolić and others [9], who demonstrated a higher ability to scavenge the stable free radical DPPH for commercially available essential oil from *G. procumbens* (IC50 30.61 mg/mL) than for the positive control of gallic acid, a strong natural antioxidant (IC50 48.52 mg/mL). Other reports documented significantly weaker effects of the oil compared to reference substances. For instance, Singh and co-workers [49] showed relatively weak antioxidant activity in the DPPH test for essential oil obtained from fresh aerial parts of eastern teaberry (EC50 94.67 μg/mL) compared to the positive standard of vitamin C (EC50 14.9 μg/mL). Similarly, Jintanasirinurak and others [47] described weak antioxidant activity of wintergreen oil obtained from leaves of *G. procumbens* in the DPPH scavenging test (IC50 > 2 × 104 ppm) and metal chelating assay (IC50 > 5 × 104 ppm), compared to the standard antioxidant vitamin C (IC50 3.16 ppm), BHT (25.23 ppm), and EDTA (18.32 ppm).

Apart from a simple DPPH test, the antioxidant activity of the wintergreen oil has been studied using several other in vitro, non-cellular and cell-based models. For instance, a commercially available essential oil from *G. procumbens* showed moderate antiradical activity, reducing the production of OH^•^ radicals in standard Fenton reaction measured by electron paramagnetic resonance spectroscopy and fluorescence assay [9]. Moreover, Eldurini and others [143] documented that adding wintergreen oil to the electrospun polycaprolactone double-layered tubular scaffolds stabilises their antioxidant capability after 8 h analysed by DPPH assay, with a slight change after 16 and 32 h. Celik and co-workers [103] demonstrated an increase in the total antioxidant capacity levels analysed by ABTS assay in cultured primary rat healthy neurons and N2a neuroblastoma cells by the essential oil (10–400 mg/L) distilled from leaves of *G. procumbens*. The same authors also showed that this essential oil did not change the total oxidative stress levels measured by ferrous ion-chelator complex assay in both cell lines at all tested concentrations [103].

The available literature usually indicates a comparable and often even higher antioxidant activity of plant extracts obtained from *G. procumbens* compared to positive controls, including powerful natural antioxidants such as quercetin or ascorbic acid, both in cellular and non-cellular tests. This activity might suggest their potential in preventing and treating diseases of affluence caused by oxidative stress. On the other hand, methyl salicylate-rich wintergreen oil presents relatively weak antioxidant activity in non-cellular in vitro models. This tendency was consistent with the reports for pure synthetic and natural salicylates, which indicated the lack of their direct free radical scavenging activity explained by the low reactivity of salicylates, including methyl salicylate as a primary aglycone, in redox reactions [73]. On the other hand, the antioxidant activity of salicylates in cellular models was proven by their significant downregulation of transcription factors, such as NF-κB, and several kinases that control the secretion of regulatory cytokines, including TNF-α [140,141]. Thus, the mechanisms of the antioxidant activity of natural salicylates, and thus of *G. procumbens*, both direct and indirect, require more profound studies and verification in vivo.

### 4.4. Photoprotective Activity

Recent studies indicated that the extracts from leaves, stems, and fruits of *G. procumbens* (5–50 μg/mL) were strong photoprotectors. Apart from the antioxidant effects revealed under UV-irradiation described above, their biological mechanism involved enhancing the viability of UVA-irradiated human Hs68 dermal fibroblasts and reducing the percentage of tail DNA in Hs68 cells as documented by CCK8 assay and the comet assay, respectively (Table 7). At the same time, the results showed that *G. procumbens* leaf, stem, and fruit extracts (5–25 μg/mL) did not affect the wound healing process, as they did not enhance fibroblast migration measured by scratch assay [89].

### 4.5. Antimicrobial, Antiparasitic, and Insecticidal Activity

The antimicrobial activity, i.e., antibacterial and antifungal, was mainly studied for the essential oil [9,10,42,43,44,47,48,49,51,52,53,55,144,145,146,147], and extracts prepared from leaves and aerial parts of *G. procumbens* [49,148,149]. The literature data also indicated the larvicidal and insecticidal activity of the essential oil [39,45,56,57,58,150,151,152,153,154,155,156,157,158,159,160,161] and plant extracts [162]. Reports on the antifungal and antibacterial activity of plant extracts and essential oils were partially divergent. Most of them suggested a weak or moderate antimicrobial activity of the wintergreen oil compared to reference substances such as streptomycin or ketoconazole, and in an individual case, even a tendency to stimulate bacterial growth [144]. In turn, available data indicated significant repellent activity and fumigant and contact toxicity of wintergreen oil against many species of insect pests, including rice weevil, wheat weevil, cowpea weevil, red flour beetle, lesser grain borer, gall midge mosquito, desert and migratory locusts, and several species of wasps among all [39,56,153,154,155,156,161,163]. The results of the antimicrobial, larvicidal, and insecticidal activity of the wintergreen oil and extracts obtained from *G. procumbens* are summarised in Table 8.

### 4.6. Human Cytochrome P450 Enzyme Inhibition

One scientific report concerned the effect of *G. procumbens* extract on inhibiting human cytochrome P450 isoenzymes. It has been shown that ethanol–water leaf extract (55:45, *v*/*v*) at 200 mg/mL revealed significant inhibitory potential towards CYP3A4 enzyme, weak on CYP19, and lack of action on CYP2C19 isoform. However, regarding the tested extract’s concentration, it was observed that its activity was about 200,000 times lower than that of the reference substance (ketoconazole). Thus, it was concluded that the eastern teaberry leaf extract has no inhibitory potential towards the metabolism of drug-specific CYPs in vivo. In addition, it was stated that it did not cause a direct change in the availability of drugs and their pharmacokinetics in the human body [164].

### 4.7. Traditional Application

Traditional medicine has been using the therapeutic effects of *G. procumbens* for hundreds of years. Preparations containing various plant parts and wintergreen oil have been and still are often used as anti-inflammatory, analgesic, and antipyretic agents for treating rheumatoid arthritis, influenza, the common cold, and fever [2,3]. In addition to medical applications, the plant material has also gained culinary recognition. Various preserves are made of fruits, and the leaves are used to prepare the warming “mountain tea” [11]. *G. procumbens* also works well as an ornamental ground cover plant due to its attractive evergreen leaves, red fruits, and significant frost resistance [12,32]. Leaf extract and wintergreen oil are frequent additives to anti-ageing cosmetics [13,165] and medical formulations for gingivitis and periodontitis [166,167,168]. Wintergreen oil is also used externally to cure lumbago, sciatica, and muscle aches [9,169,170]. 

#### 4.7.1. Historical Outline of the Origin

*G. procumbens* was known and used by the indigenous people of the North American continent, who probably noticed its characteristic smell and sumptuous, red, berry-like fruits. The Chipewyan, Iroquois, Menominee, Pottawatomi, and Senek Indian tribes used *G. procumbens* as a food source and an antipyretic and anti-rheumatic drug. The Colonists quickly noticed the delicately fragrant shrub and adapted it as a medicinal plant. The generic name *Gaultheria* was given in 1748 in honour of the French physician and researcher of Quebec, Jean Francois Gaultier (1708–1756) [171]. The first records of the cultivation of this creeping shrub come from the second half of the 18th century. Constantine S. Rafinesque (1783–1840), a well-known researcher of North American flora and fauna, described in his works the beneficial effects of *G. procumbens* and its use in restoring “vitality”, increasing lactation, treating “weakness”, asthma, and diarrhoea [172]. John King described infusions as a pain reliever after tooth extraction [6]. John M. Scudder, in turn, proposed a recipe for preparing a tincture of fresh plant material, which was supposed to soothe the bladder, prostate, and urethra, reducing irritation and inflammation [7,8]. *G. procumbens* herb and roots have been widely used in many preparations. Swaim’s Panacea was the most popular at the end of the 19th century. At the beginning of the 20th century, wintergreen oil gained recognition as a corrigent in preparations such as emulsions containing, among others, codfish liver oil [173]. Decoctions, oil solutions, or tinctures used in various inflammatory ailments were also very popular [37,174,175,176,177,178,179].

In 1820, the monograph of the *G. procumbens* leaf was introduced into the United States Pharmacopeia Supplementary List, on which the plant material remained until 1830, when its official monograph was included in the New York and Philadelphia Pharmacopoeia editions. Until 1890, the *G. procumbens* leaf functioned as an official medicine. After that time, the monograph of the plant material was initially replaced with that of the essential oil and then in 1910 with the methyl salicylate monograph [178].

*G. procumbens* was brought to Europe at the beginning of the 20th century, most likely due to its ornamental value. It is now cultivated all over the world as a ground cover plant. Evergreen shrubs look particularly beautiful during the fruiting period, from early autumn to late spring.

#### 4.7.2. Culinary Application

Raw plant materials, especially fruits of *G. procumbens*, are used as food products in countries where it is naturally occurring. The fruit is eaten raw, used as cake additives, or processed into jams, jellies, and syrups. Strong tea called “mountain tea”, candies, and wine are made from light red leaves, while very young top leaf buds are eaten raw [11]. On the other hand, the essential oil obtained from eastern teaberry is used as a food flavouring agent [9]. In the United States, it is a popular ingredient in soft drinks, such as root beer and soda [180].

#### 4.7.3. Cosmetic Application in Skin and Periodontal Ailments

The wintergreen oil and eastern teaberry leaf extract have been used for many years as ingredients for regenerating cosmetics and preparations for pain and inflammatory diseases of the mouth and gums. *G. procumbens* leaf extract (CAS number: 90045-28-6) is a cosmetic ingredient with perfuming, skin conditioning, invigorating, revitalising, and toning benefits. It is the cosmetic ally of irregular, dull, and oily skin with blemishes, which effectively restores mature skin and smooths wrinkles and fine lines. Because of its purifying and exfoliating potential, *G. procumbens* leaf extract removes excess skin sebum and improves cellular renewal. It is a popular ingredient of skin care products for anti-ageing, anti-wrinkle, resurfacing, peeling, and radiance, like facial moisturisers, masks, serums, essences, hand and around-eye creams, and scrubs [13,165].

Due to its significant anti-inflammatory activity, wintergreen oil is used to soak cotton rolls and apply them in the mouth to treat sore teeth and swelling of the gums [166]. In the appropriate dilution, the essential oil is used as a mouth rinse in treating periodontal diseases, including reducing oral cavity infections and toothache, dental caries, as well as gingivitis and periodontitis [167,168,181].

#### 4.7.4. Decorative Features

*G. procumbens* is a suitable representative of ground cover plants. The advantages of their cultivation include the ease of producing stolons, fast-spreading, and turfing of large areas, because of which the plant is successfully used to regenerate fire-damaged areas [182,183,184,185,186,187,188,189]. *G. procumbens* planted on the slopes of the hills strengthens them and protects them from washing away the soil in periods of heavy rainfall. The plant is also resistant to frost and does not require protection against the cold. Evergreen shrubs are lovely during the fruiting period, from autumn to late spring. They blend in perfectly with other plants, and the intensely red fruit, long-lasting on the plant, is a delicacy for birds [12,31,32].

## 5. Methodology

The literature search was conducted using well-known databases (Scopus, Web of Science, ScienceDirect, Google Scholar, and PubMed) using the following keyword combination pattern: (1) the species Latin or common names “*Gaultheria procumbens*”, or “American wintergreen”, or “eastern teaberry”; (2) description of the plant part/product (i.e., “whole plant”, or “aerial part/-s”, or “leaf/leaves”, or “stem/-s”, or “fruit/-s”, or “flower/-s”, or “essential oil”, or “wintergreen oil”, or “extract/-s”, or “infusion/-s”, or “decoction/-s”); (3) names of individual compounds or groups of phenolic or polyphenolic or triterpene or steroid constituents (i.e., “chemical composition”, or “chemical profile”, “qualitative profile”, or “quantitative profile”, or “salicylates”, or “methyl salicylate glycosides”, or “phenolic acids”, or “chlorogenic acids”, or “flavonoids”, or “flavonoid glycosides”, or “flavonoid aglycones”, or “catechins”, or “flavan-3-ol derivatives”, or “proanthocyanidins, or “procyanidins”, or “triterpenes”, or “sterols”, or “gaultherin”, or “methyl salicylate”, or “chlorogenic acid”, or “miquelianin”, or “epicatechin”, or “procyanidin B2”, or “cinnamtannin B1”, or “ursolic acid”, or “oleanolic acid”, or “β-sitosterol); (4) activity descriptor (i.e., “ in vitro”, or “in vivo”, or “ex vivo”, or “anti-inflammatory”, or “antioxidant”, or “photoprotective”, or “antimicrobial”, or “antibacterial”, or “antifungal”, or “larvicidal”, or “insecticidal”, or “repellent”, or “fumigant”, or “contact”, or “activity”, or “toxicity”, or “case reports”, or “inflammation”, or “oxidative stress”, or “photoprotection”); (5) medical or traditional application descriptor (i.e., “medical”, or “traditional”, or “historical”, or “culinary”, or “cosmetic”, or “periodontal”, or “decorative”). The databases were searched for original articles written in English and published (at least electronically) until November 2023. The validation of the items was performed manually (by reading the entire article), and articles with a significant contribution to the field of research are included in the present review.

## 6. Summary and Conclusions

The data reported in this review presented the current state of knowledge about the chemical composition and biological activity of various plant substances and wintergreen oil obtained from different *G. procumbens* organs. The reviewed papers covered over 180 years of phytochemical and biological studies on the qualitative and quantitative profile of the volatile fraction (essential oil) and the non-volatile lipophilic and hydrophilic components of leaves, stems, fruits, and aerial parts (herb) obtained from eastern teaberry. Over 150 studies concerned the anti-inflammatory, antioxidant, and photoprotective potential of the extracts prepared from various plant organs and the wintergreen oil’s antibacterial, antifungal, larvicidal, and insecticidal activity. Most reports covered in vitro and ex vivo non-cellular and cell-based tests. The in vivo studies only applied to the biological activity of isolated compounds, i.e., gaultherin. This work also contained an overview of available case reports of wintergreen oil accidental or intentional (suicide attempts) poisoning, with the first case recorded in 1832.

Research has shown that the aerial parts, including leaves, stems, and fruits of the evergreen shrub *G. procumbens*, are available practically throughout the whole growing season, and the autumn months (September and October) are the most favourable time for harvesting the plant material. As for chemical constituents responsible for the biological activity of eastern teaberry, the reviewed papers indicated primarily polyphenols (e.g., methyl salicylate glycosides, free flavan-3-ols and procyanidins, flavonoids, and phenolic acids with dominant isomers of chlorogenic acids), but also triterpenes, including ursolic and oleanolic acids. The available results showed that extracts from leaves, stems, and fruits of *G. procumbens* were strong anti-inflammatory and antioxidant agents that inhibited pro-inflammatory enzymes, scavenged free radicals in vitro, and downregulated the pro-inflammatory functions of human cells ex vivo. The results also revealed significant photoprotective activity of the plant extracts mentioned above. The biological results showed low in vitro antioxidant activity, moderate antibacterial and antifungal potential, and significant insecticidal and larvicidal activity of the wintergreen oil, which might be a potential candidate for an eco-friendly and effective insecticide. The data also showed the promising application of eastern teaberry leaf extract and wintergreen oil as cosmetic ingredients for several skin and periodontal ailments. Finally, the perspectives for various health-promoting uses of *G.procumbens* organs and their extracts require further studies, especially complementary and in-depth in vivo research concerning effective dose determination, drug formulation, and toxicological verification, which could constitute the basis for their broader application in official medicine and disease prevention caused by inflammation and oxidative stress.

## Figures and Tables

**Table 7:** Anti-inflammatory, antioxidant, and photoprotective activity of *G. procumbens* extracts and individual compounds.

Plant Part	Method/Activity Results	Positive Control	References
anti-inflammatory activity			
leaves	HYAL; MED: % of inhibition of the enzyme activity 2.2% (extract concentration 50 μg/mL), 4.2% (100 μg/mL); DEF: 2.1% (50 μg/mL), 4.1% (100 μg/mL); EAF: 2.5% (50 μg/mL), 21.8% (100 μg/mL); BF: 3.1% (50 μg/mL), 17.9% (100 μg/mL); WR: 0.3% (50 μg/mL), 1.9% (100 μg/mL); LOX; MED: results calculated per dw of the extract; IC_50_ = 147.4 μg/mL; DEF: IC_50_ = 200.3 μg/mL; EAF: IC_50_ = 85.4 μg/mL; BF: IC_50_ = 98.9 μg/mL; WR: IC_50_ = 323.7 μg/mL	HYAL; HP: 17.7% (50 μg/mL), 28.0% (100 μg/mL); LOX; QU: IC_50_ = 70.2 μg/mL	[68]
	results calculated per dw of the extract: HYAL; ME: IC_50_ = 18.66 μg/mL; EAE: IC_50_ = 34.57 μg/mL; BE: IC_50_ = 14.63 μg/mL LOX; ME: IC_50_ = 351.55 μg/mL; EAE: IC_50_ = 626.25 μg/mL; BE: IC_50_ = 267.04 μg/mL COX-2; ME: IC_50_ = 711.08 μg/mL; EAE: IC_50_ = 1416.93 μg/mL; BE: IC_50_ = 970.64 μg/mL	HYAL; IND: IC_50_ = 12.68 μg/mL; DEX: 14.07 μg/mL; LOX; IND: IC_50_ = 91.89 μg/mL; DEX: 120.16 μg/mL; COX-2; IND: IC_50_ = 184.32 μg/mL; DEX: 511.23 μg/mL	[60]
	LPS/*f*MLP+cytochalasin B-stimulated human neutrophils IL-8; ME: % of inhibition 4.5% (extract concentration 50 μg/mL), 11.2% (100 μg/mL), 22.6% (150 μg/mL); IL-1β; ME: 15.5% (50 μg/mL), 26.3% (100 μg/mL), 38.7% (150 μg/mL); TNF-α; ME: 4.3% (50 μg/mL), 8.7% (100 μg/mL), 19.1% (150 μg/mL); MMP-9; ME: 7.8% (50 μg/mL), 15.8% (100 μg/mL), 24.1% (150 μg/mL); ELA-2; ME: 11.7% (50 μg/mL), 22.9% (100 μg/mL), 42.3% (150 μg/mL)	IL-8; DEX: 55.8% (25 μM); IL-1β; DEX: 51.8% (25 μM); TNF-α; DEX: 68.8% (25 μM); MMP-9; DEX: 26.9% (25 μM); ELA-2; QU: 39.3% (25 μM)	
	LPS-stimulated human Hs68 dermal fibroblasts IL-8; ME: % of inhibition 1.2% (extract concentration 5 μg/mL), 22.2% (10 μg/mL), 63.8% (25 μg/mL), 69.1% (50 μg/mL); ICAM-1; ME: % of inhibition 7.9% (extract concentration 5 μg/mL), 21.5% (10 μg/mL), 30.3% (25 μg/mL), 31.8% (50 μg/mL); NF-κB; ME: % of inhibition 3.5% (extract concentration 5 μg/mL), 10.3% (10 μg/mL), 17.5% (25 μg/mL), 24.8% (50 μg/mL); phosphorylation of Erk kinase (pErk); ME: suppression of the LPS-induced Erk kinase activation (25–50 μg/mL)	IL-8; DEX: 92.7% (5 μg/mL), 95.9% (10 μg/mL), 97.9% (25 μg/mL), 99.2% (50 μg/mL); ICAM-1; DEX: 91.4% (5 μg/mL), 99.1% (10 μg/mL), 99.1% (25 μg/mL), 99.1% (50 μg/mL); NF-κB; DEX: 91.1% (5 μg/mL), 92.0% (10 μg/mL), 94.3% (25 μg/mL), 97.8% (50 μg/mL); phosphorylation of Erk kinase (pErk); DEX: suppression of the LPS-induced Erk kinase activation (25–50 μg/mL)	[89]
stems	results calculated per dw of the extract: HYAL; AE: IC_50_ = 11.67 μg/mL; ME: IC_50_ = 10.26 μg/mL; BE: IC_50_ = 15.09 μg/mL; WE: IC_50_ = 19.11 μg/mL LOX; AE: IC_50_ = 0.29 mg/mL; ME: IC_50_ = 0.32 mg/mL; BE: IC_50_ = 0.38 mg/mL; WE: IC_50_ = 0.37 mg/mL COX-2; AE: IC_50_ = 0.38 mg/mL; ME: IC_50_ = 0.47 mg/mL; BE: IC_50_ = 0.44 mg/mL; WE: IC_50_ = 0.82 mg/mL	HYAL; IND: IC_50_ = 12.77 μg/mL; DEX: 14.18 μg/mL; LOX; IND: IC_50_ = 0.09 mg/mL; DEX: 0.12 mg/mL; COX-2; IND: IC_50_ = 0.18 mg/mL; DEX: 0.51 mg/mL	[59]
	LPS/*f*MLP+cytochalasin B-stimulated human neutrophils IL-8; AE: % of inhibition 0% (extract concentration 25 μg/mL), 2.3% (50 μg/mL), 10.7% (100 μg/mL), 23.8% (150 μg/mL); IL-1β; AE: 44.2% (25 μg/mL), 62.0% (50 μg/mL), 71.3% (100 μg/mL), 81.3% (150 μg/mL); TNF-α; AE: 2.4% (25 μg/mL), 17.7% (50 μg/mL), 40.7% (100 μg/mL), 57.7% (150 μg/mL); MMP-9; AE: 2.0% (25 μg/mL), 6.2% (50 μg/mL), 13.7% (100 μg/mL), 19.5% (150 μg/mL); ELA-2; AE: 46.4% (25 μg/mL), 56.2% (50 μg/mL), 62.9% (100 μg/mL), 68.4% (150 μg/mL)	IL-8; DEX: 55.8% (25 μM), 67.8% (50 μM), 76.9% (75 μM); IL-1β; DEX: 51.8% (25 μM), 74.4% (50 μM), 79.6% (75 μM); TNF-α; DEX: 68.8% (25 μM), 91.4% (50 μM), 98.7% (75 μM); MMP-9; DEX: 26.9% (25 μM), 30.2% (50 μM), 43.7% (75 μM); ELA-2; QU: 39.3% (25 μM), 47.0% (50 μM), 55.6% (75 μM)	
	LPS-stimulated human Hs68 dermal fibroblasts IL-8; AE: % of inhibition 0.4% (extract concentration 5 μg/mL), 39.8% (10 μg/mL), 76.0% (25 μg/mL), 77.2% (50 μg/mL); ICAM-1; AE: % of inhibition 32.7% (extract concentration 5 μg/mL), 42.8% (10 μg/mL), 42.4% (25 μg/mL), 42.6% (50 μg/mL); NF-κB; ME: % of inhibition 5.9% (extract concentration 5 μg/mL), 17.7% (10 μg/mL), 20.7% (25 μg/mL), 29.1% (50 μg/mL); phosphorylation of Erk kinase (pErk); ME: suppression of the LPS-induced Erk kinase activation (25–50 μg/mL)	IL-8; DEX: 92.7% (5 μg/mL), 95.9% (10 μg/mL), 97.9% (25 μg/mL), 99.2% (50 μg/mL); ICAM-1; DEX: 91.4% (5 μg/mL), 99.1% (10 μg/mL), 99.1% (25 μg/mL), 99.1% (50 μg/mL); NF-κB; DEX: 91.1% (5 μg/mL), 92.0% (10 μg/mL), 94.3% (25 μg/mL), 97.8% (50 μg/mL); phosphorylation of Erk kinase (pErk); DEX: suppression of the LPS-induced Erk kinase activation (25–50 μg/mL)	[89]
fruits	results calculated per dw of the extract: HYAL; AE: IC_50_ = 28.39 μg/mL; ME: IC_50_ = 32.72 μg/mL; BE: IC_50_ = 49.30 μg/mL; WE: IC_50_ = 39.34 μg/mL; LOX; AE: IC_50_ = 644.79 μg/mL; ME: IC_50_ = 743.61 μg/mL; BE: IC_50_ = 850.42 μg/mL; WE: IC_50_ = 837.96 μg/mL; COX-2; AE: IC_50_ = 152.89 μg/mL; ME: IC_50_ = 230.83 μg/mL; BE: IC_50_ = 224.08 μg/mL; WE: IC_50_ = 713.36 μg/mL	HYAL; IND: IC_50_ = 12.77 μg/mL; DEX: 14.18 μg/mL; LOX; IND: IC_50_ = 92.60 μg/mL; DEX: 118.14 μg/mL; COX-2; IND: IC_50_ = 178.40 μg/mL; DEX: 507.63 μg/mL	[61]
	LPS/*f*MLP+cytochalasin B-stimulated human neutrophils IL-8; AE: % of inhibition 0% (extract concentration 25 μg/mL), 5.3% (50 μg/mL), 13.8% (100 μg/mL), 36.2% (150 μg/mL); IL-1β; AE: 28.0% (25 μg/mL), 44.7% (50 μg/mL), 63.9% (100 μg/mL), 79.7% (150 μg/mL); TNF-α; AE: 12.5% (25 μg/mL), 34.0% (50 μg/mL), 46.6% (100 μg/mL), 55.8% (150 μg/mL); MMP-9; AE: 0.6% (25 μg/mL), 5.2% (50 μg/mL), 8.5% (100 μg/mL), 17.9% (150 μg/mL); ELA-2; AE: 72.5% (25 μg/mL), 76.4% (50 μg/mL), 77.1% (100 μg/mL), 81.4% (150 μg/mL)	IL-8; DEX: 55.8% (25 μM); IL-1β; DEX: 51.8% (25 μM); TNF-α; DEX: 68.8% (25 μM); MMP-9; DEX: 26.9% (25 μM); ELA-2; QU: 39.3% (25 μM)	
	LPS-stimulated human Hs68 dermal fibroblasts IL-8; AE: % of inhibition 6.6% (extract concentration 5 μg/mL), 63.4% (10 μg/mL), 84.8% (25 μg/mL), 88.1% (50 μg/mL); ICAM-1; AE: % of inhibition 28.4% (extract concentration 5 μg/mL), 29.1% (10 μg/mL), 32.1% (25 μg/mL), 30.5% (50 μg/mL); NF-κB; ME: % of inhibition 14.5% (extract concentration 5 μg/mL), 21.8% (10 μg/mL), 29.1% (25 μg/mL), 38.7% (50 μg/mL); phosphorylation of Erk kinase (pErk); ME: suppression of the LPS-induced Erk kinase activation (25–50 μg/mL)	IL-8; DEX: 92.7% (5 μg/mL), 95.9% (10 μg/mL), 97.9% (25 μg/mL), 99.2% (50 μg/mL); ICAM-1; DEX: 91.4% (5 μg/mL), 99.1% (10 μg/mL), 99.1% (25 μg/mL), 99.1% (50 μg/mL); NF-κB; DEX: 91.1% (5 μg/mL), 92.0% (10 μg/mL), 94.3% (25 μg/mL), 97.8% (50 μg/mL); phosphorylation of Erk kinase (pErk); DEX: suppression of the LPS-induced Erk kinase activation (25–50 μg/mL)	[89]
model compounds	HYAL; QU: IC_50_ = 101.84 μM; MQ: IC_50_ = 98.15 μM; DGQ: IC_50_ = 98.08 μM; ECA: IC_50_ = 81.85 μM; PB2: IC_50_ = 37.42 μM; CB1: IC_50_ = 37.69 μM; CHA: IC_50_ = 80.69 μM; GT: IC_50_ = 64.02 μM; COX-2; QU: IC_50_ = 1.56 mM; MQ: IC_50_ = 1.29 mM; DGQ: IC_50_ = 1.44 mM; ECA: IC_50_ = 1.62 mM; PB2: IC_50_ = 1.43 mM; CB1: IC_50_ = 1.56 mM; CHA: IC_50_ = 2.86 mM; GT: IC_50_ = 0.78 mM	HYAL; IND: IC_50_ = 35.69 μM; DEX: 36.13 μM; COX-2; IND: IC_50_ = 0.50 mM; DEX: 1.29 mM	[62]
	LPS/*f*MLP+cytochalasin B-stimulated human neutrophils IL-1β; QU: % of inhibition 22.5% (compound concentration 25 μM), 44.1% (50 μM), 59.5% (75 μM); MQ: 32.9% (25 μM), 52.9% (50 μM), 66.8% (75 μM); DGQ: 42.1% (25 μM), 59.9% (50 μM), 72.3% (75 μM); ECA: 59.2% (25 μM), 67.9% (50 μM), 72.6% (75 μM); PB2: 35.1% (25 μM), 47.6% (50 μM), 64.4% (75 μM); CB1: 19.6% (25 μM), 31.9% (50 μM), 53.5% (75 μM); CHA: 47.7% (25 μM), 60.5% (50 μM), 76.2% (75 μM); GT: 13.7% (25 μM), 32.4% (50 μM), 46.6% (75 μM); TNF-α; QU: 11.1% (25 μM), 29.4% (50 μM), 55.6% (75 μM); MQ: 17.7% (25 μM), 40.4% (50 μM), 68.8% (75 μM); DGQ: 24.8% (25 μM), 61.9% (50 μM), 80.7% (75 μM); ECA: 23.4% (25 μM), 56.4% (50 μM), 72.4% (75 μM); PB2: 16.5% (25 μM), 39.9% (50 μM), 62.1% (75 μM); CB1: 7.8% (25 μM), 26.7% (50 μM), 55.8% (75 μM); CHA: 13.7% (25 μM), 50.2% (50 μM), 73.8% (75 μM); GT: 6.0% (25 μM), 48.9% (50 μM), 73.7% (75 μM); ELA-2; MQ: 38.7% (25 μM), 46.3% (50 μM), 56.2% (75 μM); DGQ: 42.5% (25 μM), 55.7% (50 μM), 66.3% (75 μM); ECA: 34.6% (25 μM), 44.8% (50 μM), 55.1% (75 μM); PB2: 40.4% (25 μM), 49.3% (50 μM), 62.4% (75 μM); CB1: 59.2% (25 μM), 66.1% (50 μM), 75.7% (75 μM); CHA: 15.5% (25 μM), 34.3% (50 μM), 43.9% (75 μM); GT: 34.1% (25 μM), 54.8% (50 μM), 64.9% (75 μM)	IL-1β; DEX: 51.8% (25 μM), 74.4% (50 μM), 79.6% (75 μM); TNF-α; DEX: 68.8% (25 μM), 91.4% (50 μM), 98.7% (75 μM); ELA-2; QU: 39.3% (25 μM), 47.0% (50 μM), 55.6% (75 μM)	
antioxidant activity			
leaves	results calculated per dw of the extract: DPPH; MEC: EC_50_ = 8.35 μg/mL, MED: 6.67 μg/mL, DEF: 4.34 μg/mL, EAF: 2.90 μg/mL, BF: 4.94 μg/mL, WR: 30.91 μg/mL; FRAP; MEC: 4.58 mmol Fe^2+^/g, MED: 5.97 mmol Fe^2+^/g, DEF: 12.50 mmol Fe^2+^/g, EAF: 12.77 mmol Fe^2+^/g, BF: 8.17 mmol Fe^2+^/g, WR: 1.46 mmol Fe^2+^/g; LA inhibition; MEC: IC_50_ = 175.98 μg/mL, MED: 207.98 μg/mL, DEF: 109.39 μg/mL, EAF: 123.94 μg/mL, BF: 164.77 μg/mL, WR: 651.85 μg/mL; O_2_^•−^; MEC: SC_50_ = 8.9 μg/mL, MED: 11.4 μg/mL, DEF: 17.4 μg/mL, EAF: 3.9 μg/mL, BF: 15.2 μg/mL, WR: 24.5 μg/mL; H_2_O_2_; MEC: SC_50_ = 10.2 μg/mL, MED: 13.7 μg/mL, DEF: 19.8 μg/mL, EAF: 7.2 μg/mL, BF: 9.2 μg/mL, WR: 19.4 μg/mL	DPPH; QU: EC_50_ = 1.63 μg/mL, TX: 4.34 μg/mL; FRAP; QU: 36.02 mmol Fe^2+^/g, TX: 10.83 mmol Fe^2+^/g; LA inhibition; QU: IC_50_ = 48.51 μg/mL, TX: 22.45 μg/mL; O_2_^•−^; AA: SC_50_ = 13.9 μg/mL; H_2_O_2_; QU: SC_50_ = 2.6 μg/mL	[68]
	ME: DPPH; results calculated per dw of the leaves; EC_50_ = 17.76 μg/mL (April), 18.17 μg/mL (May), 17.79 μg/mL (June), 17.34 μg/mL (July), 16.02 μg/mL (August), 15.00 μg/mL (September), 16.66 μg/mL (October); FRAP; results calculated per dw of the leaves; 2.44 mmol Fe^2+^/g (April), 2.33 mmol Fe^2+^/g (May), 2.38 mmol Fe^2+^/g (June), 2.53 mmol Fe^2+^/g (July), 2.62 mmol Fe^2+^/g (August), 3.41 mmol Fe^2+^/g (September), 3.29 mmol Fe^2+^/g (October)	DPPH; QU: EC_50_ = 1.63 μg/mL, TX: 4.34 μg/mL; FRAP; QU: 36.02 mmol Fe^2+^/g, TX: 10.83 mmol Fe^2+^/g	[67]
	results calculated per dw of the extract: DPPH; ME: EC_50_ = 6.77 μg/mL, EAE: 14.17 μg/mL, BE: 8.33 μg/mL; FRAP; ME: 6.36 mmol Fe^2+^/g, EAE: 3.82 mmol Fe^2+^/g, BE: 4.41 mmol Fe^2+^/g; TBARS; ME: IC_50_ = 8.46 μg/mL, EAE: 14.71 μg/mL, BE: 10.68 μg/mL; O_2_^•−^; ME: SC_50_ = 26.33 μg/mL, EAE: 39.30 μg/mL, BE: 62.36 μg/mL; OH^•^; ME: SC_50_ = 152.04 μg/mL, EAE: 480.77 μg/mL, BE: 236.51 μg/mL; H_2_O_2_; ME: SC_50_ = 44.41 μg/mL, EAE: 83.32 μg/mL, BE: 43.25 μg/mL	DPPH; QU: EC_50_ = 1.52 μg/mL, TX: 4.23 μg/mL; FRAP; QU: 49.05 mmol Fe^2+^/g, TX: 12.56 mmol Fe^2+^/g; TBARS; QU: IC_50_ = 1.69 μg/mL, TX: 4.58 μg/mL; O_2_^•−^; QU: SC_50_ = 7.35 μg/mL, TX: 142.15 μg/mL; OH^•^; QU: SC_50_ = 41.07 μg/mL, TX: 172.26 μg/mL; H_2_O_2_; QU: SC_50_ = 6.96 μg/mL, TX: 15.76 μg/mL	[60]
	*f*MLP-stimulated human neutrophils ROS; ME: level of ROS 75.9% (extract concentration 50 μg/mL), 21.5% (100 μg/mL), 5.7% (150 μg/mL)	ROS; QU: 0.8% (25 μM)	
	methanolic dry extract: DPPH: IC_50_ = 16.39 ppm; NBT/XO: 10.04%; DCF/AAPH: 9.79%	AA; DPPH: IC_50_ = 5.16 ppm, NBT/XO: 22.29%, DCF/AAPH: 20.32%; TX; DPPH: IC_50_ = 8.97 ppm, NBT/XO: 5.49%, DCF/AAPH: 24.16%; QU; DPPH: IC_50_ = 6.09 ppm, NBT/XO: -, DCF/AAPH: -	[130]
	statistical analysis of diabetic symptoms treated with *G. procumbens* leaves by indigenous peoples (Iroquois, Ojibwa, Algonquin, Cree): back pain/kidneys, blood purifier/blood tonic, rheumatism/arthritis, headache, general medicine/physic	−	[131]
	ethanol–water (90:10, *v*/*v*) extract from dry leaves: DPPH: 87.69%; ORAC: 1200 μM TX/g dw of the extract; MDA: Caco-2 cells stimulated with H_2_O_2_ with the extract at a concentration of 25 μg/mL: after 24 h (113.1%), after 48 h (89.3%); with the extract at a concentration of 100 μg/mL: after 24 h (71.4%), after 48 h (107.1%)	DPPH: −; ORAC: −; MDA: control—Caco-2 cells stimulated with H_2_O_2_ without the tested extract: after 24 h (100%), after 48 h (100%)	[88]
	UVA-irradiated human Hs68 dermal fibroblasts ROS; ME: level of ROS 79.3% (extract concentration 5 μg/mL), 67.6% (10 μg/mL), 51.9% (25 μg/mL); SOD; ME: % of enzyme activity 104.7% (extract concentration 5 μg/mL), 116.3% (10 μg/mL), 124.1% (25 μg/mL); GST; ME: % of enzyme activity 108.8% (extract concentration 5 μg/mL), 122.0% (10 μg/mL), 131.8% (25 μg/mL)	ROS; QU: 49.3% (25 μg/mL); AA: 58.6% (25 μg/mL); SOD; QU: 141.2% (25 μg/mL); AA: 148.2% (25 μg/mL); GST; QU: 113.9% (25 μg/mL); AA: 119.9% (25 μg/mL)	[89]
stems	results calculated per dw of the extract: DPPH; ME: EC_50_ = 6.42 μg/mL, BE: 6.62 μg/mL, AE: 5.67 μg/mL, WE: 8.90 μg/mL; FRAP; ME: 6.01 mmol Fe^2+^/g, BE: 6.41 mmol Fe^2+^/g, AE: 7.65 mmol Fe^2+^/g, WE: 5.45 mmol Fe^2+^/g; TBARS; ME: IC_50_ = 7.15 μg/mL, BE: 12.12 μg/mL, AE: 6.70 μg/mL, WE: 15.50 μg/mL; O_2_^•−^; ME: SC_50_ = 26.54 μg/mL, BE: 34.49 μg/mL, AE: 22.44 μg/mL, WE: 25.48 μg/mL; OH^•^; ME: SC_50_ = 152.79 μg/mL, BE: 178.96 μg/mL, AE: 149.24 μg/mL, WE: 153.66 μg/mL; H_2_O_2_; ME: SC_50_ = 34.79 μg/mL, BE: 38.28 μg/mL, AE: 33.01 μg/mL, WE: 56.41 μg/mL	DPPH; QU: EC_50_ = 1.65 μg/mL, TX: 4.31 μg/mL; FRAP; QU: 47.09 mmol Fe^2+^/g, TX: 11.89 mmol Fe^2+^/g; TBARS; QU: IC_50_ = 1.78 μg/mL, TX: 4.68 μg/mL; O_2_^•−^; QU: SC_50_ = 7.58 μg/mL, TX: 135.24 μg/mL; OH^•^; QU: SC_50_ = 42.48 μg/mL, TX: 165.45 μg/mL; H_2_O_2_; QU: SC_50_ = 7.52 μg/mL, TX: 15.87 μg/mL	[59]
	*f*MLP-stimulated human neutrophils model ROS; AE: level of ROS 49.7% (extract concentration 25 μg/mL), 28.8% (50 μg/mL), 10.5% (100 μg/mL), 6.2% (150 μg/mL)	ROS; QU: 17.8% (25 μM), 7.8% (50 μM), 0.8% (75 μM)	
	UVA-irradiated human Hs68 dermal fibroblasts ROS; AE: level of ROS 82.0% (extract concentration 5 μg/mL), 73.8% (10 μg/mL), 58.4% (25 μg/mL); SOD; AE: % of enzyme activity 114.4% (extract concentration 5 μg/mL), 124.5% (10 μg/mL), 139.9% (25 μg/mL); GST; AE: % of enzyme activity 104.6% (extract concentration 5 μg/mL), 113.6% (10 μg/mL), 125.4% (25 μg/mL)	ROS; QU: 49.3% (25 μg/mL); AA: 58.6% (25 μg/mL); SOD; QU: 141.2% (25 μg/mL); AA: 148.2% (25 μg/mL); GST; QU: 113.9% (25 μg/mL); AA: 119.9% (25 μg/mL)	[89]
fruits	results calculated per dw of the extract: DPPH; ME: EC_50_ = 40.40 μg/mL, BE: 237.00 μg/mL, AE: 44.59 μg/mL, WE: 75.46 μg/mL; FRAP; ME: 1.65 mmol Fe^2+^/g, BE: 0.93 mmol Fe^2+^/g, AE: 1.75 mmol Fe^2+^/g, WE: 0.99 mmol Fe^2+^/g; TBARS; ME: IC_50_ = 56.67 μg/mL, BE: 251.68 μg/mL, AE: 37.41 μg/mL, WE: 71.84 μg/mL; O_2_^•−^; ME: SC_50_ = 333.41 μg/mL, BE: 877.63 μg/mL, AE: 175.33 μg/mL, WE: 322.94 μg/mL; OH^•^; ME: SC_50_ = 824.04 μg/mL, BE: 676.29 μg/mL, AE: 863.95 μg/mL, WE: 1150.67 μg/mL; H_2_O_2_; ME: SC_50_ = 332.15 μg/mL, BE: 497.95 μg/mL, AE: 166.36 μg/mL, WE: 422.95 μg/mL	DPPH; QU: EC_50_ = 1.65 μg/mL, TX: 4.31 μg/mL; FRAP; QU: 47.09 mmol Fe^2+^/g, TX: 11.89 mmol Fe^2+^/g; TBARS; QU: IC_50_ = 1.78 μg/mL, TX: 4.68 μg/mL; O_2_^•−^; QU: SC_50_ = 7.58 μg/mL, TX: 135.24 μg/mL; OH^•^; QU: SC_50_ = 42.48 μg/mL, TX: 165.45 μg/mL; H_2_O_2_; QU: SC_50_ = 7.52 μg/mL, TX: 15.87 μg/mL	[61]
	*f*MLP-stimulated human neutrophils model ROS; AE: level of ROS 83.8% (extract concentration 25 μg/mL), 72.1% (50 μg/mL), 55.2% (100 μg/mL), 35.5% (150 μg/mL)	ROS; QU: 17.8% (25 μM)	
	dry extracts prepared with an aqueous citric acid solution at pH = 2.0: DPPH; 87.55% (1–15 October 2006), 89.28% (2–30 October 2006), 85.88% (3–15 March 2007)	−	[30]
	DPPH; CAE: 89.2%, liquid methanol–water extract (80:20, *v*/*v*): 92.3%; ABTS; CAE: 47.7%, liquid methanol–water extract (80:20, *v*/*v*): 46.2%	−	[72]
	UVA-irradiated human Hs68 dermal fibroblasts ROS; AE: level of ROS 95.5% (extract concentration 5 μg/mL), 90.9% (10 μg/mL), 78.3% (25 μg/mL); SOD; AE: % of enzyme activity 102.6% (extract concentration 5 μg/mL), 110.3% (10 μg/mL), 120.9% (25 μg/mL); GST; AE: % of enzyme activity 107.6% (extract concentration 5 μg/mL), 116.9% (10 μg/mL), 127.8% (25 μg/mL)	ROS; QU: 49.3% (25 μg/mL); AA: 58.6% (25 μg/mL); SOD; QU: 141.2% (25 μg/mL); AA: 148.2% (25 μg/mL); GST; QU: 113.9% (25 μg/mL); AA: 119.9% (25 μg/mL)	[89]
model compounds	FRAP; QU: 14.23 mol/mol, MQ: 9.24 mol/mol, DGQ: 6.39 mol/mol, ECA: 10.39 mol/mol, PB2: 17.11 mol/mol, CB1: 16.30 mol/mol, CHA: 9.06 mol/mol, GT: 0.29 mol/mol; O_2_^•−^; QU: SC_50_ = 25.08 μM, MQ: 32.55 μM, DGQ: 18.00 μM, ECA: 7.89 μM, PB2: 6.26 μM, CB1: 5.31 μM, CHA: 19.72 μM, GT: 1012.00 μM	FRAP; AA: 3.97 mol/mol, TX: 2.98 mol/mol; O_2_^•−^; AA: SC_50_ = 29.87 μM, TX: 540.33 μM	[62]
	*f*MLP-stimulated human neutrophils ROS; MQ: level of ROS 71.1% (compound concentration 25 μM), 54.7% (50 μM), 36.8% (75 μM); DGQ: 78.9% (25 μM), 65.6% (50 μM), 42.6% (75 μM); ECA: 27.1% (25 μM), 14.9% (50 μM), 8.1% (75 μM); PB2: 41.7% (25 μM), 30.6% (50 μM), 23.4% (75 μM); CB1: 78.7% (25 μM), 54.1% (50 μM), 37.3% (75 μM); CHA: 32.6% (25 μM), 23.0% (50 μM), 17.6% (75 μM); GT: 78.5% (25 μM), 55.2% (50 μM), 47.7% (75 μM)	ROS; QU: 48.2% (25 μM), 25.6% (50 μM), 20.4% (75 μM)	
photoprotective activity			
leaves	UVA-irradiated human Hs68 dermal fibroblasts cell viability after UVA-irradiation; ME: % of viable cells 105.2% (extract concentration 5 μg/mL), 110.4% (10 μg/mL), 115.4% (25 μg/mL); DNA damage; ME: % of tail DNA 54.6% (5 μg/mL), 51.2% (10 μg/mL), 40.7% (25 μg/mL), 30.8% (50 μg/mL)	cell viability after UVA-irradiation; QU: 115.6% (25 μg/mL); AA: 120.4% (25 μg/mL); DNA damage; QU: 27.2% (25 μg/mL); AA: 33.9% (25 μg/mL)	[89]
stems	UVA-irradiated human Hs68 dermal fibroblasts cell viability after UVA-irradiation; AE: % of viable cells 109.8% (extract concentration 5 μg/mL), 115.9% (10 μg/mL), 119.8% (25 μg/mL); DNA damage; AE: % of tail DNA 62.1% (5 μg/mL), 55.3% (10 μg/mL), 45.3% (25 μg/mL), 32.1% (50 μg/mL)		
fruits	UVA-irradiated human Hs68 dermal fibroblasts cell viability after UVA-irradiation; AE: % of viable cells 99.5% (extract concentration 5 μg/mL), 105.3% (10 μg/mL), 109.3% (25 μg/mL); DNA damage; AE: % of tail DNA 63.4% (5 μg/mL), 49.7% (10 μg/mL), 38.6% (25 μg/mL), 33.9% (50 μg/mL)		

AA: ascorbic acid; DEX: dexamethasone; IND: indomethacin; HP: heparin; QU: quercetin; TX: Trolox^®^ ((±)-6-hydroxy-2,2,7,8-tetramethylchroman-2-carboxylic acid); MEC/ME: methanol–water dry extract (75:25, *v*/*v*) obtained by direct extraction of the raw material with a solvent; MED: defatted methanol–water extract (75:25, *v*/*v*) obtained by preliminary extraction of the raw material with chloroform in a Soxhlet apparatus, followed by extraction with a methanol–water solution (75:25, *v*/*v*); DEF: diethyl ether fraction (fractionated extraction); EAF: ethyl acetate fraction (fractionated extraction); BF: *n*-butanol fraction (fractionated extraction); WR/WF: water residue/fraction (fractionated extraction); ME: methanol–water dry extract (75:25, *v*/*v*); EAE: ethyl acetate dry extract (direct extraction); BE: *n*-butanol dry extract (direct extraction); AE: acetone dry extract (direct extraction); WE: water dry extract (direct extraction); CAE: liquid extract prepared with 0.1 M aqueous citric acid; tests measuring the level of cytokine and pro-inflammatory enzymes release and ROS level by stimulated with LPS (bacterial lipopolysaccharide obtained from *Escherichia coli* O111:B4), *f*MLP (*N*-formyl-L-methionyl-L-leucyl-L-phenylalanine) or *f*MLP + cytochalasin B human neutrophils isolated from buffy coats, including IL-8: interleukin 8; IL-1β: interleukin 1β; TNF-α: tumour necrosis factor α (tumour necrosis factor α); MMP-9: matrix methylproteinase 9; ELA-2: human elastase type 2; ICAM-1: intercellular adhesion molecule 1; NF-κB: nuclear factor kappa-light-chain-enhancer of activated B cells; Erk: extracellular signal-regulated kinase; ROS: reactive oxygen species; SOD: superoxide dismutase; GST: glutathione S-transferase; HYAL: hyaluronidase inhibition assay; LOX: lipoxygenase inhibition assay; COX-2: cyclooxygenase 2 inhibition assay; DPPH: DPPH^•^ radical scavenging test; FRAP: antioxidant activity expressed in millimoles of Fe^2+^ ions/g of tested extract or reference substance (ferric-reducing antioxidant power); LA inhibition: linoleic acid oxidation inhibition test; O_2_^•−^: superoxide radical anion scavenging test; H_2_O_2_: hydrogen peroxide reduction test; TBARS: linoleic acid oxidation inhibition test that measures the level of thiobarbituric acid-reactive substances produced; OH^•^: hydroxyl radical scavenging test; NBT/XO: hypoxanthine/xanthine oxidase-generated superoxide anion scavenging test in the presence of nitrotetrazolium blue; DCF/AAPH: 2,7-dichlorofluorescein diacetate fluorimetric test for scavenging peroxyl radicals (ROO^•^) resulting from the thermal decomposition of AAPH (2,2-azobis-(2-amidinopropane) dihydrochloride); ORAC: fluorimetric test for scavenging of peroxyl radicals (ROO^•^) formed as a result of thermal decomposition of AAPH (oxygen radical absorbance capacity); MDA: malonyldialdehyde assay using thiobarbituric acid and analysed on the Caco-2 cell line (Caucasian colon adenocarcinoma immortalised human colon adenocarcinoma cell line); ABTS: ABTS^•+^ cation scavenging test; IC_50_: concentration of a tested extract or reference substance, expressed in μg/mL or mg/mL, which reduces the enzyme activity or inhibits the oxidation of linolenic acid by 50% (*inhibitory concentration*); EC_50_: effective concentration of a tested extract or reference substance, expressed in μg/mL or mg/mL, which causes a 50% decrease in the radical concentration (*effective concentration*); SC_50_: concentration of a tested extract or reference substance, expressed in μg/mL or mg/mL, which causes a 50% radical scavenging (*scavenging concentration*).

**Table 8:** Antimicrobial, larvicidal, and insecticidal activity of essential oil and extracts of *G. procumbens*.

Essential Oil/Extract	Microorganism/Activity Results	Positive Control	References
antibacterial activity			
essential oil from herbs (aerial parts)	microdilution method (%): *Acinetobacter baumannii* (MIC = 0.25%, *v*/*v*), *Aeromonas sobria* (0.25%), *Enterococcus faecalis* (>2.0%), *Escherichia coli* (0.5%), *Klebsiella pneumoniae* (1.0%), *Pseudomonas aeruginosa* (>2.0%), *Salmonella typhimurium* (0.5%), *Serratia marcescens* (0.5%), *Staphylococcus aureus* (2.0%)	−	[145]
	disc diffusion method; zone of inhibition diameter (mm): *Staphylococcus aureus*: 19.5 mm (essential oil concentration: 0.1%, *v*/*v*), 20.4 mm (0.5%), 22.0 mm (1.0%), *Escherichia coli*: 18.2 mm (0.1%), 18.8 mm (0.5%), 19.9 mm (1.0%)	chloramphenicol (0.1 mg/mL): *S. aureus:* 23.2 mm, *E. coli:* 21.9 mm	[49]
essential oil from leaves	disc diffusion method; zone of inhibition diameter (mm): *Staphylococcus aureus* (7.33 mm), *Staphylococcus capitis* (8.33 mm), *Staphylococcus epidermidis* (4.33–5.67 mm), *Staphylococcus haemoliticus* (4.33–6.33 mm), *Staphylococcus hominis* (3.33–5.67 mm) microdilution method (μL/mL): *Staphylococcus aureus* (MIC = 12.50 μL/mL), *Staphylococcus capitis* (12.50 μL/mL), *Staphylococcus epidermidis* (25.00 μL/mL), *Staphylococcus haemoliticus* (12.50–25.00 μL/mL), *Staphylococcus hominis* (25.00 μL/mL)	−	[44]
	microdilution method (mg/mL): *Escherichia coli* (MIC = 6.33 mg/mL), *Aeromonas caviae* (3.16 mg/mL), *Serratia marcescens* (6.33 mg/mL), *Staphylococcus aureus* (6.33 mg/mL), *Streptococcus pneumoniae* (12.67 mg/mL), *Candida albicans* (1.82 mg/mL), *Candida tropicalis* (7.29 mg/mL), *Candida glabrata* (7.29 mg/mL), *Mycobacterium fortuitum* (3.64 mg/mL), *Mycobacterium abscessus* (3.64 mg/mL)	−	[42]
	disc diffusion method; zone of inhibition diameter (mm): *Staphylococcus aureus* (23.9 mm), *Escherichia coli* (7.9 mm)	amoxicillin (50 μg/mL): *S. aureus* (33.8 mm), *E. coli* (19.2 mm)	[47]
	microdilution method (MIC and MBC in % essential oil): *Pasteurella multocida* (MIC = 0.5%; MBC not possible to determine); disc diffusion method: median inhibition zone radius (mm); interquartile range: *Pasteurella multocida* (14.5 mm; 1.25); *Mannheimia haemolytica* (15.5 mm); *Mannheimia* clade (12 mm)	growth control (with only sterile blank filter paper discs); negative control (wintergreen oil without bacterial inoculum)	[48]
	microdilution method (mg/mL): *Staphylococcus aureus* (MIC = 14.0 mg/mL), *Enterococus faecalis* (16.4 mg/mL), *Escherichia coli* (9.4 mg/mL), *Acinetobacter baumanii* (8.2 mg/mL)	gentamicin: *S. aureus* (MIC = 0.016 mg/mL), *E. faecalis* (0.064 mg/mL), *E. coli* (0.004 mg/mL), *A. baumanii* (0.032 mg/mL)	[10]
	clinical strains and environmental bacteria microdilution method (mg/mL): *Staphylococcus aureus* (pus, MIC = 14.1 mg/mL), *Staphylococcus aureus* (pharynx, 14.1 mg/mL), *Enterococcus faecalis* (urine, 16.7 mg/mL), *Enterococcus faecalis* (respiratory, 16.1 mg/mL), *Escherichia coli* (wound, 10.0 mg/mL), *Escherichia coli* (bronchia, 8.8 mg/mL), *Acinetobacter baumanii* (sputum, 8.5 mg/mL), *Acinetobacter baumanii* (sink, 8.5 mg/mL)	−	
essential oil from fruits	microdilution method (mg/mL): *Staphylococcus aureus* (MIC = 13.5 mg/mL), *Enterococus faecalis* (15.3 mg/mL), *Escherichia coli* (8.8 mg/mL), *Acinetobacter baumanii* (8.2 mg/mL)	gentamicin: *S. aureus* (MIC = 0.016 mg/mL), *E. faecalis* (0.064 mg/mL), *E. coli* (0.004 mg/mL), *A. baumanii* (0.032 mg/mL)	
	clinical strains and environmental bacteria; microdilution method (mg/mL): *Staphylococcus aureus* (pus, MIC = 14.1 mg/mL), *Staphylococcus aureus* (pharynx, 13.2 mg/mL), *Enterococcus faecalis* (urine, 15.5 mg/mL), *Enterococcus faecalis* (respiratory, 16.4 mg/mL), *Escherichia coli* (wound, 9.7 mg/mL), *Escherichia coli* (bronchia, 8.8 mg/mL), *Acinetobacter baumanii* (sputum, 8.8 mg/mL), *Acinetobacter baumanii* (sink, 8.5 mg/mL)	−	
commercial essential oil	microdilution method (mg/mL): *Listeria monocytogenes* (MIC = 0.29 mg/mL), *Staphylococcus aureus* (0.36 mg/mL), *Escherichia coli* (0.36 mg/mL), *Pseudomonas aeruginosa* (0.18 mg/mL), *Salmonella typhimurium* (0.36 mg/mL)	streptomycin: *L. monocytogenes* (MIC = 0.05 mg/mL), *S. aureus* (0.05 mg/mL), *E. coli* (0.20 mg/mL), *P. aeruginosa* (0.05 mg/mL), *S. typhimurium* (0.10 mg/mL)	[9]
	growth (%) of bacteria in the tested sample (milk + bacteria + essential oil at a concentration 4%, *v*/*v*): *Staphylococcus aureus*: 108% *Staphylococcus chromogenes*: 65% *Streptococcus uberis*: 159%	control: sample containing milk + bacteria: 100% bacterial growth	[144]
	*Pseudominas tolaasii* (MIC = 0.08 µg/mL; MBC = 0.16 µg/mL), *Clavibacter michiganensis* subsp*. michiganensis* (MIC = 0.08 µg/mL; MBC = 0.16 µg/mL), *Xanthomonas campestris* pv*. phaseoli* (MIC = 0.02 µg/mL; MBC = 0.02 µg/mL)	−	[53]
	microdilution method (μL/mL): *Aerococcus* spp. (MIC = 12.5 μL/mL); *Aerococcus viridans* (6.25 μL/mL); *Aeromonas* spp. (3.12 μL/mL*); Aeromonas bestiarum* (6.25 μL/mL); *Aeromonas salmonicida* (3.12 μL/mL); *Escherichia vulgaris* (25.0 μL/mL); *Enterococcus faecium* (3.12 μL/mL); *Enterococcus moravensis* (3.12 μL/mL); *Enterococcus aquimarinus* (3.12 μL/mL); *Pseudomonas fluorescens* (25.0 μL/mL); *Pseudomonas frederiksbergensis* (25.0 μL/mL); *Pseudomonas gessardii* (25.0 μL/mL); *Pseudomonas ludensis* (25.0 μL/mL); *Pseudomonas proteolitica* (6.25 μL/mL); *Shewanella baltica* (6.25 μL/mL); *Yersinia enterocolitica* (6.25 μL/mL); *Yersinia ruckeri* (6.25 μL/mL); *Yersinia* spp. (6.25 μL/mL); *Vagococcus* spp. (6.25 μL/mL)	−	[55]
ethanol–water (55:45, *v*/*v*) extract from leaves	*Neisseria gonorrhoeae*MIC: >512 µg/mL	penicillin G: MIC = 128 µg/mL	[148]
alcoholic extract from herb (aerial parts)	concentration 5% (*w*/*v*) disc diffusion method; zone of inhibition diameter (mm): *Staphylococcus aureus*: 20.8 mm, *Escherichia coli*: 19.0 mm	chloramphenicol (0.1 mg/mL): *S. aureus:* 23.2 mm, *E. coli:* 21.9 mm	[49]
antifungal activity			
essential oil from herb (aerial parts)	*Candida albicans* (MIC = 0.25%, *v*/*v*)	−	[145]
	disc diffusion method; zone of inhibition diameter (mm): *Candida albicans*: 16.5 mm (essential oil concentration 0.1%, *v*/*v*), 17.2 mm (0.5%), 18.0 mm (1.0%); *Aspergillus niger*: 15.9 mm (0.1%), 16.6 mm (0.5%), 17.2 mm (1.0%)	ketoconazole (0.1 mg/mL): *C. albicans* (18.8 mm), *A. niger* (18.1 mm)	[49]
essential oil from leaves	disc diffusion method; zone of inhibition diameter (mm): *Torulaspora delbruecki* (32.66 mm), *Debaromyces hanseni* spp*. hanseni* (29.65 mm), *Kluyveromyces maxianus* spp*. lactis* (36.54 mm), *Pichia polymorpha* (40.23 mm), *Candida lactis* spp*. condensi* (14.23 mm), *Candida humulis* (16.74 mm), *Saccharomyces cerevisiae* (16.90 mm), *Zygosaccharomyces lactis* (9.67 mm), *Zygosaccharomyces pombe* (8.80 mm), *Torula lactis* (15.81 mm) microdilution method (μg/mL): *Torulaspora delbruecki* (MIC = 8.79 μg/mL; MFC = 5.86 μg/mL), *Debaromyces hanseni* spp*. hanseni* (17.58 μg/mL; 11.72 μg/mL), *Kluyveromyces maxianus* spp*. lactis* (5.85 μg/mL; 2.93 μg/mL), *Pichia polymorpha* (4.39 μg/mL; 19.53 μg/mL), *Candida lactis* spp*. condensi* (29.30 μg/mL; 17.58 μg/mL), *Candida humulis* (35.16 μg/mL; 23.44 μg/mL), *Saccharomyces cerevisiae* (58.60 μg/mL; 35.16 μg/mL), *Zygosaccharomyces lactis* (562.50 μg/mL; 78.13 μg/mL), *Zygosaccharomyces pombe* (375.00 μg/mL; 156.25 μg/mL), *Torula lactis* (234.38 μg/mL; 78.13 μg/mL)	−	[43]
commercial essential oil	microdilution method (mg/mL): *Aspergillus flavus* (MIC = 2.92 mg/mL), *Aspergillus fumigatus* (0.73 mg/mL), *Aspergillus niger* (0.73 mg/mL), *Aspergillus ochraceus* (1.46 mg/mL), *Penicillium funiculosum* (0.73 mg/mL), *Penicillium ocrochloron* (1.46 mg/mL)	ketoconazole: *A. flavus* (1.50 mg/mL), *A. fumigatus* (0.20 mg/mL), *A. niger* (0.20 mg/mL), *A. ochraceus* (0.20 mg/mL), *P. funiculosum* (0.20 mg/mL), *P. ocrochloron* (1.00 mg/mL)	[9]
	disc diffusion method; zone of inhibition diameter (cm): *Phomopsis azadirachtae:* 7.00 cm (essential oil concentration 500 ppm), 5.40 cm (1000 ppm), 0.00 cm (1500 ppm), 0.00 cm (2000 ppm), 0.00 cm (2500 ppm)	Tween 20: 9.00 cm	[146]
	antifungal activity against 11 plant pathogens	−	[147]
	*Trichophyton mentagrophytes* disc diffusion method; zone of inhibition diameter (mm): 0 mm box vapour assay: MFD > 100 μg/mL	−	[52]
	*Fusarium*, including *F. verticilliodies*, *F. napiforme*, and *F. delphinoides* MIC: 2400–38,400 µg/mL	natamycin: MIC: 2–256 µg/mL	[51]
alcoholic extract from herb (aerial parts)	concentration 5% (*w*/*v*) disc diffusion method; zone of inhibition diameter (mm): *Candida albicans*: 17.0 mm, *Aspergillus niger*: 16.8 mm	ketoconazole (0.1 mg/mL): *C. albicans* (18.8 mm), *A. niger* (18.1 mm)	[49]
ethanol–water (1:1, *v*/*v*) extract from leaves	disc diffusion method; zone of inhibition diameter (mm): *Microsporum gypseum:* 7.9 mm, *Trichophyton mentagrophytes:* 7.7 mm; the extract did not work on: *Saccharomyces cerevisiae*, *Cryptococcus neoformans*, *Candida albicans,* and *Aspergillus fumigatus*	berberine (2 mg): *M. gypseum:* 18.0 mm, *T. mentagrophytes:* 20.0 mm, *S. cerevisiae:* 10.0 mm, *C. neoformans:* 26.0 mm, *C. albicans:* 16.0 mm, *A. fumigatus:* 10.0 mm	[149]
larvicidal, insecticidal, and other activity			
essential oil from leaves	*Cortaderia selloana* (pampas grass): in vitro effect against seed germination: 83% (concentration of wintergreen oil 0.125 μL/mL), 77% (0.25 μL/mL), 56% (0.5 μL/mL), 19% (1 μL/mL); *Nicotiana glauca* (tree tobacco): in vitro effect against seed germination: 90% (concentration of wintergreen oil 0.125 μL/mL), 87% (0.25 μL/mL), 91% (0.5 μL/mL), 79% (1 μL/mL)	control: untreated seedlings *C. selloana*: 84%; *N. glauca*: 94%	[45]
	influenza A/WS/33 virus lack of anti-influenza activity (no reduction in visible cytopathic effects of influenza A/WS/33 virus activity) at a concentration of 100 μg/mL	control: oseltamivir (100 μg/mL): 49%	[150]
	*Sitophilus oryzae* (rice weevil) and *Rhyzopertha dominica* (lesser grain borer) method: jars with wheat seeds, paper discs treated with wintergreen oil (without any solvents), and 50 adults (4–6 days old) of both insects; antifeedant activity: *S. oryzae*: % seed damage (0.0%), % weight loss (0.0%), FDI (100%), *R. dominica*: % seed damage (0.0%), % weight loss (0.0%), FDI (100%); fumigant toxicity: *S. oryzae*: LC_50_ = 58.62 μL/L (51–85–64.47 μL/L), *R. dominica:* LC_50_ = 2.71 μL/L (2.36–3.03 μL/L)	control: paper discs without wintergreen oil and any solvents; antifeedant activity: *S. oryzae*: % seed damage (65.04%), % weight loss (8.26%), FDI (0%); *R. dominica*: % seed damage (76.04%), % weight loss (5.33%), FDI (0%)	[39]
	*Callosobruchus chinensis* (pulse beetle, Chinese bruchid, cowpea bruchid) method: glass cylinder with paper discs treated with wintergreen oil dissolved in acetone; fumigant toxicity after 24 h: LC_50_ = 3.14 mg/L	control: paper discs treated with acetone	[151]
commercial essential oil	fourth instar larvae of *Agrotis ipsilon* (dark sword-grass moth) method: antifeedant activity; Petri dishes with fresh potato leaf discs treated with wintergreen oil dissolved in water and one 2 h pre-starved insect; method: insecticidal activity; round plastic trough with fresh potato leaf discs treated with wintergreen oil dissolved in water and 10 2 h pre-starved insects; antifeedant activity: 19.79% (wintergreen oil concentration 0.25%), 28.41% (0.5%), 54.66% (1.0%), 87.21% (2.0%); insecticidal activity: 2.70% (0.25%), 18.41% (0.5%), 53.64% (1.0%), 86.92% (2.0%)	control: leaf discs treated with water; antifeedant activity: 4.22%, insecticidal activity: 1.20%	[152]
	third instar larvae of *Camptomyia corticalis* (Cecidomyiidae gall midge, mosquito) method: Petri dishes with paper discs treated with wintergreen oil dissolved in ethanol and 20 insects; % mortality after 24 h: 100% (wintergreen oil concentration 1.41 mg/cm^3^), 97% (1.05 mg/cm^3^); fumigant toxicity during a 24 h exposure: LC_50_ = 0.74 mg/cm^3^ (0.65–0.87 mg/cm^3^)	control: paper discs treated with ethanol; positive control: dichlorvos—conventional insecticide fumigant toxicity: LC_50_ = 0.027 mg/cm^3^ (0.023–0.032 mg/cm^3^)	[153]
	fourth instar larvae of *Tribolium castaneum* (red flour beetle) method: Petri dishes with paper discs treated with wintergreen oil dissolved in water and 10 insects; repellent activity: 21.2% (wintergreen oil concentration 5 μL/mL), 43.04% (10 μL/mL), 65.96% (15 μL/mL), 80.2% (20 μL/mL); larvicidal activity after 96 h: 18.4% (5 μL/mL), 37.78% (10 μL/mL), 54.64% (15 μL/mL), 69.91% (20 μL/mL)	control: paper discs treated with water; repellent activity: 0.0%; larvicidal activity after 24 h: 0.0% (not tested after 96 h)	[154]
	*Callosobruchus maculatus* (cowpea weevil, cowpea seed beetle) method: fumigant toxicity; airtight plastic jars with 20 cowpea seeds, paper discs treated with wintergreen oil dissolved in acetone, and four newly emerged *C. maculatus*; fumigant toxicity: wintergreen oil concentration 5 μL/mL, mortality after 6 h (10%), after 12 h (60%), after 24 h (85%), and 48 h (93.33%); wintergreen oil concentration 10 μL/mL, mortality after 6 h (15%), after 12 h (70%), after 24 h (90%), and 48 h (100%); wintergreen oil concentration 20 μL/mL, mortality after 6 h (45%), after 12 h (75%), after 24 h (100%), and 48 h (100%); wintergreen oil concentration 40 μL/mL, mortality after 6 h (80%), after 12 h (100%), after 24 h (100%), and 48 h (100%) method: ovicidal activity; plastic jars with 20 cowpea seeds, paper discs treated with wintergreen oil dissolved in acetone, and 20 eggs of *C. maculatus*; ovicidal activity during 48 h of exposure: wintergreen oil concentration 5 μL/mL (51.16%), wintergreen oil concentration 10 μL/mL (63.95%), wintergreen oil concentration 20 μL/mL (93.02%), wintergreen oil concentration 40 μL/mL (100.00%)	control: paper discs treated with acetone	[155]
	wasps: *Vespula pensylvanica* (western yellowjacket), *Vespula vulgaris* (common wasp), *Vespula germanica* (German yellowjacket), *Dolichovespula maculate* (black-faced hornet), *Polistes dominula* (European paper wasp) and *Paralucia aurifera* (golden paper wasp) field experiment (traps set in nature); capture of social wasps in Rescue!^®^ repellent release rate: attractant (acetic acid+2-methyl-1-butanol)+essential oil (45 mg/day); mean per trap per visit: Yellowjackets (*Vespula pensylvanica*, *V. vulgaris*, *V. germanica*, *Dolichovespula maculata*) = 2.3; Paper wasps (*Polistes dominulus*, *Paralucia aurifer*) = 2.0	control: repellent release rate: attractant without wintergreen oil (0 mg/day); mean per trap per visit: Yellowjackets = 43.7, Paper wasps = 7.9	[156]
	*Sitophylus granarius* (wheat weevil) method: Falcon tubes containing wheat treated with wintergreen oil dissolved in acetone and 20 insects; mortality (%) after 24 h of exposure: wintergreen oil concentration 5% (100%), 4% (−), 3% (96%), 2% (81%), 1% (5%)	control: wheat treated with acetone; mortality (%) after 24 h of exposure: 0%	[56]
	*Anarsia lineatella* (peach twig borer, fruit moth) method: ULV (Ultra Low Volumes)-olfactory apparatus with peach twigs having one fruit and 2–5 leaves sprayed with wintergreen oil dissolved in water; the number of eggs laid by females of *A. lineatella*: 59.8 eggs; longevity of females of *A. lineatella*: 15.4 days; the number of eggs laid per day by female fruit moths: after 5 days (6.4 eggs), 10 days (23.6 eggs), 15 days (31.2 eggs)	control: peach twigs sprayed with distilled water, soybean oil, and emulsifier—Tween 20; the number of eggs laid by females of *A. lineatella*: 128 eggs; longevity of females of *A. lineatella*: 23.2 days; the number of eggs laid per day by female fruit moths: after 5 days (7.4 eggs), 10 days (27.4 eggs), 15 days (32.6 eggs)	[157]
	three weeks *Arabidopsis thaliana* Col-0 plants (thale cress) wintergreen oil sprayed on leaves: β-glucuronidase activity (~52 pKata/mg protein); a strong expression of a selection of defence genes detected 1, 6, and 24 h after wintergreen oil treatment using a high-throughput qPCR-based microfluidic method; induction of plant resistance by wintergreen oil (1 μL/mL) sprayed on leaves following inoculation with a conidial suspension of a GFP-expressing strain of the *Arabidopsis thaliana* fungal pathogen *Colletotrichum higginsianum* 48 h later: strong reduction (~60%) of pathogen development: Relative Fluorescence Units (~397 RFU)	control: wetting agent (benzo(1,2,3)-thiadiazole-7-carbothiolic acid (BTH)): β-glucuronidase activity (~0.8 pKata/mg protein); methyl salicylate: β-glucuronidase activity (~47 pKata/mg protein); methyl salicylate: strong expression of a selection of defence genes; wetting agent: ~985 RFU; commercial product; BION^®^ (40 μg/mL): ~309 RFU	[158]
	*Paederus fuscipes* (rove beetle) method: repellent activity; Petri dishes with half filter paper discs treated with wintergreen oil dissolved in acetone and 10 insects; fumigant toxicity; filter paper treated with wintergreen oil; contact toxicity; wintergreen oil dissolved in acetone applied topically to the dorsal thorax of the beetles; contact activity: wintergreen oil concentration 0.08 μL/adult; corrected mortality after 1 h (40.00%), after 2 h (66.67%), after 4 h (73.33%), after 6 h (80.00%), after 8 h (82.22%); contact toxicity: LC_50_ = 0.086 μL/adult (after 1 h of exposure), LC_50_ = 0.067 μL/adult (after 2 h of exposure), LC_50_ = 0.062 μL/adult (after 4 h of exposure), LC_50_ = 0.061 μL/adult (after 6 h of exposure), LC_50_ = 0.060 μL/adult (after 8 h of exposure); fumigant activity: wintergreen oil concentration 2 μL/L air; corrected mortality after 1 h (24.44%), after 2 h (35.56%), after 4 h (46.67%), after 6 h (68.89%), after 8 h (80.00%); fumigant toxicity: LC_50_ = 2.680 μL/L air (after 1 h of exposure), LC_50_ = 2.178 μL/L air (after 2 h of exposure), LC_50_ = 2.046 μL/L air (after 4 h of exposure), LC_50_ = 1.765 μL/L air (after 6 h of exposure), LC_50_ = 1.591 μL/L air (after 8 h of exposure); repellent activity: wintergreen oil concentration 0.1 μL/cm^2^; repellency after 1 h (97.78%), after 2 h (−%), after 4 h (−%), after 6 h (−%), after 8 h (−%); repellent activity: wintergreen oil concentration 0.01 μL/cm^2^; repellency after 1 h (30.00%), after 2 h (18.89%), after 4 h (12.22%), after 6 h (10.00%), after 8 h (4.44%)	control: paper discs treated with acetone (repellent activity); glass vial with filter paper untreated with wintergreen oil (fumigant toxicity); acetone applied topically on the beetles (contact toxicity)	[58]
	*Paederus fuscipes* (rove beetle) method: wintergreen oil dissolved in acetone and acetylcholinesterase activity analysed in vitro: *P. fuscipes* male adults: 13.0252 nM/min per mg—specific activity and 21.20% inhibition rate of enzyme activity (wintergreen oil concentration: 0.909 μL/L air), 11.0914 nM/min per mg—specific activity and 32.91% inhibition rate of enzyme activity (wintergreen oil concentration: 1.273 μL/L air), 10.9243 nM/min per mg—specific activity and 33.78% inhibition rate of enzyme activity (wintergreen oil concentration: 1.636 μL/L air); *P. fuscipes* female adults: 17.5222 nM/min per mg—specific activity and 17.87% inhibition rate of enzyme activity (wintergreen oil concentration: 0.909 μL/L air), 13.8935 nM/min per mg—specific activity and 35.57% inhibition rate of enzyme activity (wintergreen oil concentration: 1.273 μL/L air), 12.2137 nM/min per mg—specific activity and 43.33% inhibition rate of enzyme activity (wintergreen oil concentration: 1.636 μL/L air); method: five insects fumigated with wintergreen oil dissolved in acetone, homogenised, and acetylcholinesterase activity analysed in vivo: *P. fuscipes* male adults in vivo: 17.4306 nM/min per mg—specific activity and 10.37% inhibition rate of enzyme activity (wintergreen oil concentration: 0.1 μL), 15.5895 nM/min per mg—specific activity and 19.76% inhibition rate of enzyme activity (wintergreen oil concentration: 0.5 μL), 14.9349 nM/min per mg—specific activity and 23.15% inhibition rate of enzyme activity (wintergreen oil concentration: 1.0 μL), 14.0898 nM/min per mg—specific activity and 27.50% inhibition rate of enzyme activity (wintergreen oil concentration: 2.0 μL), 13.4049 nM/min per mg—specific activity and 31.06% inhibition rate of enzyme activity (wintergreen oil concentration: 3.0 μL); *P. fuscipes* female adults in vivo: 18.9940 nM/min per mg—specific activity and 20.75% inhibition rate of enzyme activity (wintergreen oil concentration: 0.1 μL), 15.8405 nM/min per mg—specific activity and 33.89% inhibition rate of enzyme activity (wintergreen oil concentration: 0.5 μL), 15.6144 nM/min per mg—specific activity and 34.87% inhibition rate of enzyme activity (wintergreen oil concentration: 1.0 μL), 15.4384 nM/min per mg—specific activity and 35.57% inhibition rate of enzyme activity (wintergreen oil concentration: 2.0 μL), 15.9878 nM/min per mg—specific activity and 33.32% inhibition rate of enzyme activity (wintergreen oil concentration: 3.0 μL)	control: in vitro (acetone instead of wintergreen oil): *P. fuscipes* male adults: 16.5564 nM/min per mg—specific activity and 0% inhibition rate of enzyme activity; *P. fuscipes* female adults: 21.5605 nM/min per mg—specific activity and 0% inhibition rate of enzyme activity; control: in vivo (acetone instead of wintergreen oil): *P. fuscipes* male adults in vivo: 19.4385 nM/min per mg—specific activity and 0% inhibition rate of enzyme activity; *P. fuscipes* female adults in vivo: 23.9783 nM/min per mg—specific activity and 0% inhibition rate of enzyme activity	[57]
	*Apis mellifera* (honey bees) mortality (%) after ingestion of syrup containing wintergreen oil and ethanol (total number of bees: 1308): 1% (day 1), 24% (day 8, concentration of wintergreen oil 1000 ppm), 99% (day 14, concentration of wintergreen oil 100 000 ppm); LD_50_ for wintergreen oil at 8 days exposure: 13,500 ppm; LD_50_ for wintergreen oil at 14 days exposure: 800 ppm	control: syrup containing ethanol	[159]
	*Loxosceles reclusa* (brown recluse spider) wintergreen oil presented the most significant mortality among all tested essential oils measured by direct contact or as a fumigant (inhalation) treatment for 24 h; the mortality in the fumigant toxicity test (<20%) was lower than in the contact toxicity assay	−	[160]
	*Schistocera gregaria* (desert locust) and *Locusta migratoria* (migratory locust) method: plastic boxes with wheat grass, sprayed with an emulsion containing linseed oil, 10% sodium bicarbonate solution, and wintergreen oil, and 10 locusts (half females and half males); mortality (%): desert locust (12%), migratory locust (50%)	control: wheat grass sprayed with pure linseed oil or 10% sodium bicarbonate solution; mortality (%): desert locust (0%), migratory locust (0%)	[161]
	method: Petri dishes with pre-cut filter paper squares sprayed with wintergreen oil dissolved in ethanol and 10 insects and clean green bean leaves; mortality (%) after 1 h of exposure to the essential oil: *Orius laevigatus*: wintergreen oil concentration 0.125 *v*/*v* (86.67%), 0.25 *v*/*v* (93.33%), 0.5 *v*/*v* (100%); toxicity category: 3 (moderately harmful); *Nesidiocoris tenuis*: wintergreen oil concentration 0.125 *v*/*v* (86.67%), 0.25 *v*/*v* (96.67%), 0.5 *v*/*v* (100%); toxicity category: 3–4 (moderately harmful to harmful); *Macrolophus pygmaeus*: wintergreen oil concentration 0.125 *v*/*v* (16.67%), 0.25 *v*/*v* (20.00%), 0.5 *v*/*v* (26.67%); toxicity category: 1 (harmless); *Encarsia formosa* (chalcidoid wasp; parasitoid of greenhouse whitefly): wintergreen oil concentration 0.125 *v*/*v* (0%), 0.25 *v*/*v* (0%), 0.5 *v*/*v* (0%); toxicity category: 1 (harmless); *Eretmocerus eremicus* (parasitoid of sweet potato whitefly): wintergreen oil concentration 0.125 *v*/*v* (0%), 0.25 *v*/*v* (0%), 0.5 *v*/*v* (0%); toxicity category: 1 (harmless); mortality (%) after 3 h of exposure to the essential oil: *Orius laevigatus*: wintergreen oil concentration 0.125 *v*/*v* (100%), 0.25 *v*/*v* (100%), 0.5 *v*/*v* (100%); toxicity category: 4 (harmful); *Nesidiocoris tenuis*: wintergreen oil concentration 0.125 *v*/*v* (100%), 0.25 *v*/*v* (100%), 0.5 *v*/*v* (100%); toxicity category: 4 (harmful); *Macrolophus pygmaeus*: wintergreen oil concentration 0.125 *v*/*v* (36.67%), 0.25 *v*/*v* (43.33%), 0.5 *v*/*v* (56.67%); toxicity category: 2 (slightly harmless); *Encarsia formosa* (chalcidoid wasp; parasitoid of greenhouse whitefly): wintergreen oil concentration 0.125 *v*/*v* (3.33%), 0.25 *v*/*v* (6.67%), 0.5 *v*/*v* (10.00%); toxicity category: 1 (harmless); *Eretmocerus eremicus* (parasitoid of sweet potato whitefly): wintergreen oil concentration 0.125 *v*/*v* (10.00%), 0.25 *v*/*v* (10.00%), 0.5 *v*/*v* (13.33%); toxicity category: 1 (harmless); mortality (%) after 24 h of exposure to the essential oil: *Orius laevigatus*: wintergreen oil concentration 0.125 *v*/*v* (-), 0.25 *v*/*v* (-), 0.5 *v*/*v* (-); toxicity category: -; *Nesidiocoris tenuis*: wintergreen oil concentration 0.125 *v*/*v* (100%), 0.25 *v*/*v* (-), 0.5 *v*/*v* (-); toxicity category: 4 (harmful); *Macrolophus pygmaeus*: wintergreen oil concentration 0.125 *v*/*v* (73.33%), 0.25 *v*/*v* (90.00%), 0.5 *v*/*v* (80.00%); toxicity category: 3–4 (moderately harmful to harmful); *Encarsia formosa* (chalcidoid wasp; parasitoid of greenhouse whitefly): wintergreen oil concentration 0.125 *v*/*v* (6.67%), 0.25 *v*/*v* (13.33%), 0.5 *v*/*v* (20.00%); toxicity category: 1 (harmless); *Eretmocerus eremicus* (parasitoid of sweetpotato whitefly): wintergreen oil concentration 0.125 *v*/*v* (16.67%), 0.25 *v*/*v* (23.33%), 0.5 *v*/*v* (26.67%); toxicity category: 1 (harmless)	control: Petri dishes with pre-cut filter paper squares sprayed with water and 10 insects and clean green bean leaves	[163]
ethanol–water (80:20, *v*/*v*), dimethylsulfoxide (DMSO), and water extracts	*Leishmania mexicana* (LM227 strain) method: dye-binding method using bovine serum albumin as a standard; % inhibition of LM227 growth per mg protein at 48 h; ethanol–water (80:20, *v*/*v*) extract: not tested; DMSO extract: <5% per mg protein; water extract: <5% per mg protein	control: extract solvent instead of plant extract	[162]

MIC: minimum inhibitory concentration; MBC: minimum bactericidal concentration; MFD: minimum fungicidal dose; FDI: feeding deterrent index.

## Data Availability

Not applicable.

## Supplementary Materials

The following supporting information can be downloaded at: https://www.mdpi.com/article/10.3390/ijms25010565/s1.

## Abbreviations

AA	ascorbic acid
AAPH	2,2′-azobis-(2-amidinopropane) dihydrochloride
ABTS	2,2′-azinobis-(3-ethylbenzothiazoline-6-sulfonic acid)
AE	acetone dry extract (direct extraction)
AG	astragalin
BE	*n*-butanol dry extract (direct extraction)
BF	*n*-butanol fraction (fractionated extraction)
β-SIT	β-sitosterol
CA	(+)-catechin
Caco-2	Caucasian colon adenocarcinoma immortalised human colon adenocarcinoma cell line
CAE	liquid extract prepared with 0.1 M aqueous citric acid
CB1	cinnamtannin B1
CHA	chlorogenic acid
CCHA	cryptochlorogenic acid
CHE	chloroform dry extract
COX-2	cyclooxygenase 2
CYE	cyanidin chloride equivalents
DCF	2,7-dichlorofluorescein diacetate
DEF	diethyl ether fraction (fractionated extraction)
DEX	dexamethasone
DGK	wintergreenoside B
DGQ	wintergreenoside A
DPPH	2,2-diphenyl-1-picrylhydrazyl (DPPH^•^) radical
dw	dry weight
EAE	ethyl acetate dry extract (direct extraction)
EAF	ethyl acetate fraction (fractionated extraction)
EC	effective concentration
ECA	(−)-epicatechin
ELA-2	neutrophils elastase 2
Erk	extracellular signal-regulated kinase
FDI	feeding deterrent index
FLC	total flavonoids determined by HPLC-PDA as sum of aglycones after acid hydrolysis
*f*MLP	*N*-formyl-L-methionyl-L-leucyl-L-phenylalanine
FRAP	ferric-reducing antioxidant power
GAE	gallic acid equivalents
GST	glutathione *S*-transferase
GT	methyl salicylate 2-*O*-β-D-xylopyranosyl-(1→6)-β-D-glucopyranoside (gaultherin)
GV	guaijaverin
H_2_O_2_	hydrogen peroxide
HP	heparin
HY	hyperoside
HYAL	hyaluronidase
IC	inhibitory concentration
IND	indomethacin
ICAM-1	intercellular adhesion molecule 1
IL	interleukin
IQ	isoquercitrin
KA	kaempferol
KG	kaempferol 3-*O*-glucuronide
LA	linoleic acid
LOX	lipoxygenase
LPS	bacterial lipopolysaccharide obtained from *Escherichia coli* O111:B4
MBC	minimum bactericidal concentration
MDA	malonyldialdehyde
ME	methanol–water dry extract (75:25, *v*/*v*)
MEC/ME	methanol–water dry extract (75:25, *v*/*v*) obtained by direct extraction of the raw material with a solvent
MED	defatted methanol–water extract (75:25, *v*/*v*) obtained by preliminary extraction of the raw material with chloroform in a Soxhlet apparatus, followed by extraction with a methanol–water solution (75:25, *v*/*v*)
MFD	minimum fungicidal dose
MIC	minimum inhibitory concentration
MMP-9	matrix metalloproteinase 9
MQ	miquelianin
NBT	nitro-blue tetrazolium chloride
NCHA	neochlorogenic acid
NF-κB	nuclear factor kappa-B
OA	oleanolic acid
O_2_^•−^	superoxide radical anion
^•^OH	hydroxyl radical
ORAC	oxygen radical absorbance capacity
PB2	procyanidin B2
PCA	protocatechuic acid
PE	petroleum ether dry extract
*p*-HBA	*p*-hydroxybenzoic acid
PH	methyl salicylate 2-*O*-β-D-glucopyranosyl-(1→2)-β-D-glucopyranoside (physanguloside A)
QCT	quercitrin
QU	quercetin
ROS	reactive oxygen species
SC	scavenging concentration
SOD	superoxide dismutase
SPHA	total content of simple phenolic acids derivatives of hydroxycinnamic and hydroxybenzoic acids determined by the HPLC-PDA-fingerprint
TBARS	thiobarbituric acid-reactive substances
TCHA	total monocaffeoylquinic acid content determined by HPLC-PDA-fingerprint
TFL	total flavonoid content determined by HPLC-PDA-fingerprint
TG	methyl salicylate 2-*O*-β-D-glucopyranosyl-(1→2)-[*O*-β-D-xylopyranosyl-(1→6)]-*O*-β-D-glucopyranoside
TLPA	total proanthocyanidin content determined by HPLC-PDA-fingerprint
TNF-α	tumour necrosis factor α
TPA	total proanthocyanidin content determined by *n*-BuOH/HCl spectrophotometric method (Porter’s method)
TPC	total phenolic content determined by Folin–Ciocalteu assay
TPH	total phenolic content determined by HPLC-PDA-fingerprint
TPHA	total content of phenolic acids determined by HPLC-PDA-fingerprint
TSAL	total salicylate content determined by HPLC-PDA-fingerprint
TX	(±)-6-hydroxy-2,2,7,8-tetramethylchroman-2-carboxylic acid (Trolox^®^)
UA	ursolic acid
WE	water dry extract (direct extraction)
WR/WF	water residue/fraction (fractionated extraction)
XO	xanthine oxidase
