# Supplementary material for: Phytochemistry and Biological Profile of Gaultheria procumbens L. and Wintergreen Essential Oil: From Traditional Application to Molecular Mechanisms and Therapeutic Targets
Source: Int J Mol Sci. 2024 Jan 1;25(1):565. doi: 10.3390/ijms25010565 (PMC10778675; doi:10.3390/ijms25010565)
Supplement: Supplementary file 1 [file ijms-25-00565-s001.zip › ijms-2762468-supplementary.pdf]

Review

Phytochemistry and Biological Profile of *Gaultheria procumbens* L. and Wintergreen Essential Oil: From Traditional Application to Molecular Mechanisms and Therapeutic Targets.

Piotr Michel, Monika Anna Olszewska *

Department of Pharmacognosy, Faculty of Pharmacy, Medical University of Lodz, Muszyńskiego 1, 90-151 Lodz, Poland; piotr.michel@umed.lodz.pl

* Correspondence: monika.olszewska@umed.lodz.pl; Tel.: +48 426779165

Supplementary Materials:

Figure S1. Simple hydroxybenzoic and hydroxycinnamic acids: *p*-hydroxybenzoic acid (S1), protocatechuic acid (S2), vanillic acid (S3), *p*-coumaric acid (S4), and caffeic acid (S5).

Figure S2. Monocaffeoylquinic acids: chlorogenic acid (S6), neochlorogenic acid (S7), and cryptochlorogenic acid (S8). The nomenclature of caffeoylquinic acid isomers is according to IUPAC [1].

Figure S3. Flavonoid aglycones: quercetin (S9), kaempferol (S10), and flavonoid monoglycosides: hyperoside (S11), isoquercitrin (S12), miquelianin (S13), guaijaverin (S14), kaempferol 3-*O*-glucuronide (S15), astragalin (S16), and quercitrin (S17).

Figure S4. Catechins: (+)-catechin (S18) and (–)-epicatechin (S19), and procyanidin dimer: procyanidin B2 (S20).

Figure S5. The dominant components of *G. procumbens* essential oils apart from methyl salicylate: *α*-pinene (S21), camphene (S22), β-pinene (S23), limonene (S24), sabinene (S25), eugenol (S26), citral (S27), β-myrcene (S28), fenchone (S29), and menthone (S30).

Figure S6. Triterpenes: oleanolic acid (S31), ursolic acid (S32), and sterols: β-sitosterol (S33).

Table S1. The chemical composition of *G. procumbens* essential oils.

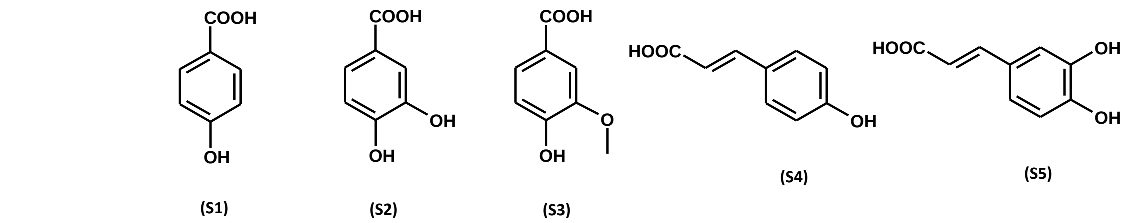

Figure S1. Simple hydroxybenzoic and hydroxycinnamic acids: *p*-hydroxybenzoic acid (S1), protocatechuic acid (S2), vanillic acid (S3), *p*-coumaric acid (S4), and caffeic acid (S5).

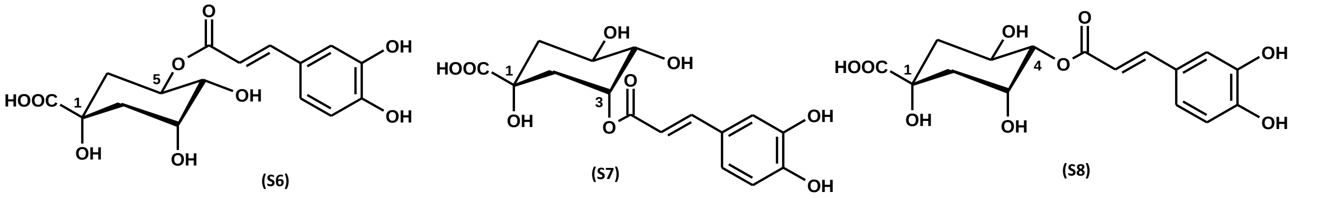

Figure S2. Monocaffeoylquinic acids: chlorogenic acid (S6), neochlorogenic acid (S7), and cryptochlorogenic acid (S8). The nomenclature of caffeoylquinic acid isomers is according to IUPAC [1].

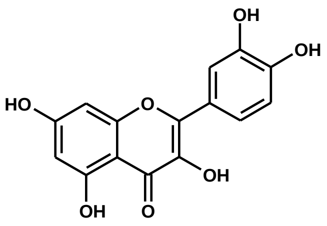

(S9)

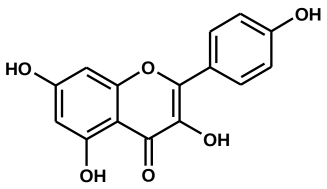

(S10)

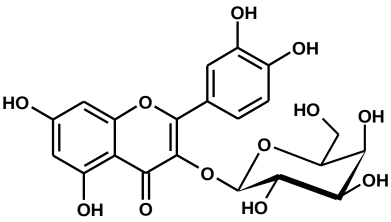

(S11)

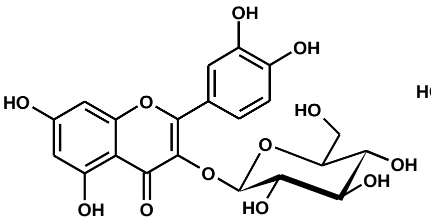

(S12)

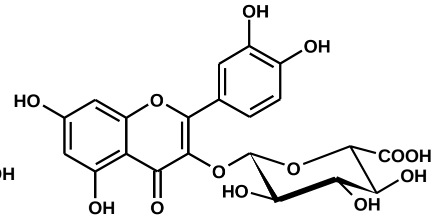

(S13)

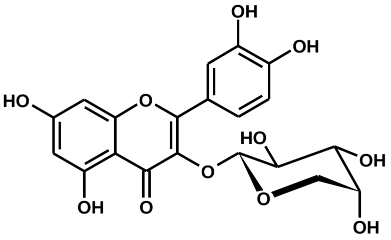

(S14)

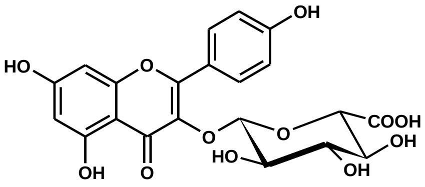

(S15)

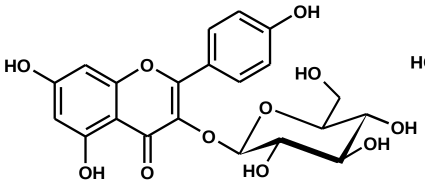

(S16)

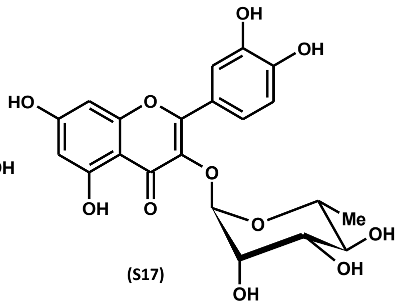

(S17)

Figure S3. Flavonoid aglycones: quercetin (S9), kaempferol (S10), and flavonoid monoglycosides: hyperoside (S11), isoquercitrin (S12), miquelianin (S13), guaijaverin (S14), kaempferol 3-*O*-glucuronide (S15), astragalin (S16), and quercitrin (S17).

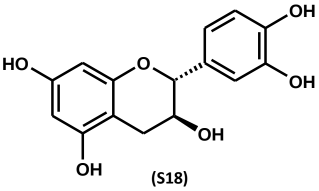

(S18)

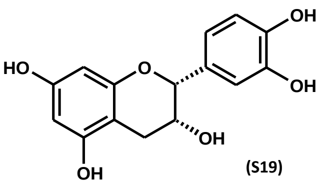

(S19)

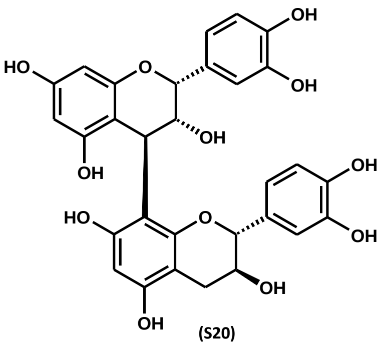

(S20)

Figure S4. Catechins: (+)-catechin (S18) and (–)-epicatechin (S19), and procyanidin dimer: procyanidin B2 (S20).

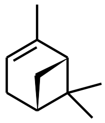

(S21)

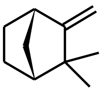

(S22)

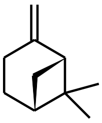

(S23)

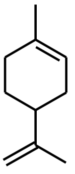

(S24)

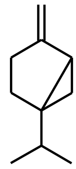

(S25)

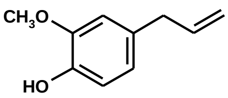

(S26)

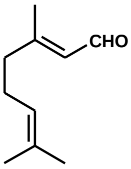

(S27)

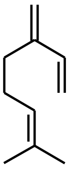

(S28)

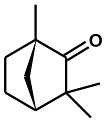

(S29)

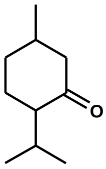

(S30)

Figure S5. The dominant components of *G. procumbens* essential oils apart from methyl salicylate: α-pinene (S21), camphene (S22), β-pinene (S23), limonene (S24), sabinene (S25), eugenol (S26), citral (S27), β-myrcene (S28), fenchone (S29), and menthone (S30).

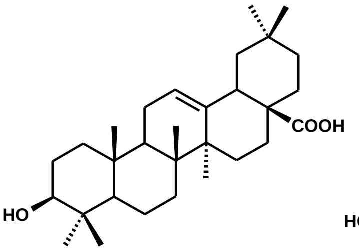

(S31)

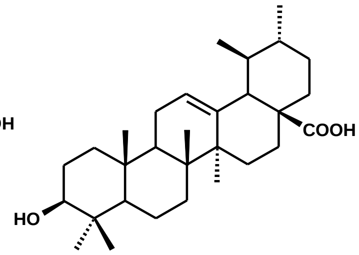

(S32)

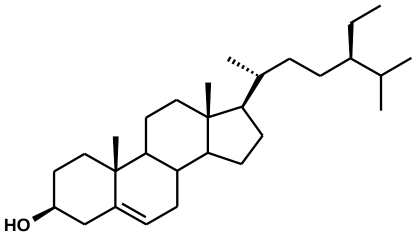

(S33)

Figure S6. Triterpenes: oleanolic acid (S31), ursolic acid (S32), and sterols: β-sitosterol (S33).

Table S1. The chemical composition of *G. procumbens* essential oils.

Compound	Fruits	Leaves							Aerial parts	Commercial essential oil		
pent-1-en-3-ol	tr											
pentanal	tr											
pent-4-enal	0.01											
pentan-1-ol	tr											
hexanal	0.03											0.00-0.01
furfural	0.03											
(*E*)-hex-2-enal		tr										0.00-0.01
4-methylhex-5-en-2-ol	0.02											
hex-3-en-1-ol	tr	tr										0.00-0.09
hex-2-en-1-ol		tr										0.00-0.01
santene		tr										
heptanal	tr											0.00-0.01
heptan-2-ol	0.07	0.02										0.00-0.04
α-pinene		0.04	0.076				0.18	0.02	2.66	0.22	0.2	0.00-0.05
(*S*)-2-ethyl-4-methyl-pentan-1-ol		tr										
hept-2-enal	tr											
camphene		tr							1.02			0.00-0.01
heptan-1-ol	tr											0.00-tr
oct-4-en-3-on	tr											
benzaldehyde	0.01											0.00-0.01
β-pinene		0.01					0.21	0.01		0.25	0.3	0.00-0.02
6-methylhept-5-en-2-ol	tr											
oct-1-en-3-ol	tr	tr										
2-penthylfuran	0.01											
octanal	tr	tr										
2-methylhept-6-en-1-ol		tr										
oct-3,5-dien-2-ol	tr											
2-ethylhexan-1-ol	tr											
limonene	0.01	0.01					2.05	0.01	5.54	2.17	2.2	0.00-0.33
p-cymene	tr	0.01										0.00-0.05
benzyl alcohol	0.55					0.2						tr-0.04

oct-2-enal	tr											
octan-1-ol	0.01	0.01										0.00-0.06
octa-3,5-dien-2-on	tr											
sabinene	tr									0.08	0.1	0.00-0.01
heptanoic acid	0.37											
nonanal	0.01					tr						
nonan-2-ol	0.01											
linalool	0.01				0.04			0.03				śl-0.11
borneol	tr											
naphthalene	0.01											
nonan-1-ol		tr										
octanoic acid	0.08											
izopinocarweol	0.01											0.00-0.01
β-cyclocitral		tr										0.00-0.01
methyl salicylate	97.47	99.76	96.61	96.25	99.96	91.1	96.87	99.63	61.14	96.90	96.9	97.36-99.71
dec-3-en-1-ol	0.01											
nonanoic acid	0.01											
p-cymene-7-ol	0.06											
bornyl acetate		tr										0.00-0.08
spiro[4,5]decane-1-one		0.03										
methyl-*o*-anisate		0.07										
eugenol	0.02							0.01	1.73			0.00-0.12
2,6-dihydroxybenzoic acid methyl ester	0.02											
neric acid	tr											
isoeugenol	0.01											
massoilactone	tr	0.04				1.3						
acetylsalicylic acid methyl ester		tr										0.00-0.01
3-hydroxy-phenylacetic acid	0.39											
tridecanedial	tr											
heptadec-13-in-1-ol	tr											
abieta-8,11,13-triene		tr										

octadeca-13,16-dienoic acid methyl ester	tr											
4-hydroxy-4-methylpentan-2-on			0.096									
δ-3-caren			0.118									
(*Z*)-citral			0.735	1.020								0.00-0.08
(*E*)-citral				1.110								0.00-0.06
citral			0.916									
diethyl phthalate			0.452	0.424								
α-thuiene									0.08			
fenchol									1.43			
p-mentha-1,3,8-triene									2.65			
cis-tagetone									6.16			
ethyl 3-octanoate									0.53			
n-butanoic acid									7.06			
7-hydroxy-3,7-dimethyloctanal									2.24			
1-ethenyl-3,5-dimethylbenzene									0.51			
Ethyl cuminaldehyde									2.08			
1,2-dihydroxybutanyl-4-1',2',4'-trimethylbenzene									0.06			
o-methylphenoxy-2,3-dimethylbenzene									2.70			
2-butenyl isopulegol									1.16			
o-methylphenoxy-2-propenyltoluene									1.25			
β-myrcene										0.09	0.1	0.00-0.01
fenchone										0.17	0.2	
menton										0.12	0.1	
ethanol												0.00-1.37
acetone												0.00-0.03
hexan-1-ol												0.00-0.04
phenol												0.00-0.02

(*Z*)-3-hexenyl acetate												0.00-0.01
α-phellandrene												0.00-0.01
α-terpinene												0.00-0.01
1,8-cineole+β-fellandrene												0.00-0.01
γ-terpinene												0.00-0.03
terpinolene												0.00-0.01
camphor								0.01				0.00-0.01
isopulegol												0.00-0.01
ethyl benzoate												0.00-0.01
terpinene-4-ol												0.00-0.02
nerol												0.00-0.05
geraniol												0.00-0.06
ethyl salicylate								0.18				0.05-0.50
safrole												0.00-0.16
methyl-*o*-cresotinate												0.00-tr
vitispirane												0.02-0.03
isobornyl acetate												0.00-0.02
nagina ketone (dehydro Elshotzia ketone)												0.00-0.01
α-terpenyl acetate												0.00-0.03
methyl 2,4-dihydroxybenzo-ate												0.00-0.01
α-copaene												0.00-0.01
(*E*)-β-caryophyllene						tr		0.01				0.00-0.04
aromadendrene												0.00-0.05
α-humulene												0.00-0.01
eugenyl acetate												0.00-0.03
δ-cadinene						tr						0.00-0.03
(*Z*)-3-hexenyl benzoate												0.00-0.01
benzyl salicylate						tr						0.00-0.05
2,3,6-trimethylhepta-1,5-diene						tr						
menthone						0.1						
coumaran						tr						

pulegone						0.6						
(2*E*)-decenal						tr						
(*E*)-cinnamaldehyde						tr						
thymol						0.3						
(*E*)-cinnamyl alcohol						0.8						
piperitenone						tr						
trans-β-elemene						tr						
trans-α-bergamotene						tr						
geranyl acetone						0.1						
tuberolactone						5.3						
germacrane D						0.1						
(*E*,*E*)-α-farnesene						tr						
caryophyllene oxide						tr						
α-cadinol						tr						
8-hydroxyisobornyl isobuta-noate						tr						
benzyl benzoate						tr						
1,8-cineole								0.03				
**References**	[2]		[3]	[4]	[5]	[6]	[7]	[8]	[9]	[10]	[11]	[12]

tr - traces < 0.01%; numerical values express the relative content of compounds as a percentage of the total content of all essential oil components.

References

1. Abrankó, L.; Clifford, M.N. An unambiguous nomenclature for the acyl-quinic acids commonly known as chlorogenic acids. *J. Agric. Food Chem.* **2017**, *65*, 3602–3608, doi:10.1021/acs.jafc.7b00729.
2. Magiera, A.; Sienkiewicz, M.; Olszewska, M.A.; Kicel, A.; Michel, P. Chemical profile and antibacterial activity of essential oils from leaves and fruits of *Gaultheria procumbens* L. cultivated in Poland. *Acta Pol. Pharm. - Drug Res.* **2019**, *76*, 93–102, doi:10.32383/appdr/94245.
3. Kiran, S.; Prakash, B. Assessment of toxicity, antifeedant activity, and biochemical responses in stored-grain insects exposed to lethal and sublethal doses of *Gaultheria procumbens* L. essential oil. *J. Agric. Food Chem.* **2015**, *63*, 10518–10524, doi:10.1021/acs.jafc.5b03797.
4. Kujur, A.; Kiran, S.; Dubey, N.K.; Prakash, B. Microencapsulation of *Gaultheria procumbens* essential oil using chitosan-cinnamic acid microgel: Improvement of antimicrobial activity, stability and mode of action. *LWT - Food Sci. Technol.* **2017**, *86*, 132–138, doi:10.1016/j.lwt.2017.07.054.
5. Verdi, C.M.; Machado, V.S.; Machado, A.K.; Klein, B.; Bonez, P.C.; de Andrade, E.N.C.; Rossi, G.; Campos, M.M.; Wagner, R.; Sagrillo, M.R.; et al. Phytochemical characterization, genotoxicity, cytotoxicity, and antimicrobial activity of *Gaultheria procumbens* essential oil. *Nat. Prod. Res.* **2022**, *36*, 1327–1331, doi:10.1080/14786419.2020.1862832.
6. Lawson, S.K.; Satyal, P.; Setzer, W.N. The volatile phytochemistry of seven native American aromatic medicinal plants. *Plants* **2021**, *10*, 1061, doi:10.3390/plants10061061.
7. Sevik, R.; Akarca, G. Investigation of the chemical composition of *Myrtus communis* L. and *Gaultheria procumbens* L. essential oils and their effects on foodborne yeasts. *J. Essent. Oil Bear. Plants* **2021**, *24*, 1146–1158, doi:10.1080/0972060X.2021.2010606.
8. Ibáñez, M.D.; Blázquez, M.A. Tea tree and wintergreen essential oils in the management of the invasive species *Cortaderia selloana* and *Nicotiana glauca*. *J. Plant Prot. Res.* **2019**, *59*, 160–169, doi:10.24425/jppr.2019.129281.
9. Singh, V.; Ali, G.M. Isolation of volatile constituents and biological studies of aerial parts of *Gaultheria procumbens* L. *Int. J. Green Pharm.* **2017**, *11*, 784–788, doi:10.22377/IJGP.V11I04.1359.
10. Nikolić, M.; Marković, T.; Mojović, M.; Pejin, B.; Savić, A.; Perić, T.; Marković, D.; Stević, T.; Soković, M. Chemical composition and biological activity of *Gaultheria procumbens* L. essential oil. *Ind. Crops Prod.* **2013**, *49*, 561–567, doi:10.1016/j.indcrop.2013.06.002.
11. Todorović, B.; Potočnik, I.; Rekanović, E.; Stepanović, M.; Kostić, M.; Ristić, M.; Milijašević-Marčić, S. Toxicity of twenty-two plant essential oils against pathogenic bacteria of vegetables and mushrooms. *J. Environ. Sci. Heal. - Part B Pestic. Food Contam. Agric. Wastes* **2016**, *51*, 832–839, doi:10.1080/03601234.2016.1208462.
12. Cuchet, A.; Jame, P.; Anchisi, A.; Schiets, F.; Oberlin, C.; Lefèvre, J.-C.; Carénini, E.; Casabianca, H. Authentication of the naturalness of wintergreen (*Gaultheria* genus) essential oils by gas chromatography, isotope ratio mass spectrometry and radiocarbon assessment. *Ind. Crops Prod.* **2019**, *142*, 111873, doi:10.1016/j.indcrop.2019.111873.
